# Novel insights into the relationship between glaucoma and brain diseases from the genetic to diseases levels: A cross-sectional study

**DOI:** 10.1097/MD.0000000000044416

**Published:** 2025-09-19

**Authors:** Xingyi Chen, Chaoran Shi, Meihui He, Xiaobo Xia

**Affiliations:** a Eye Center of Xiangya Hospital, Central South University, Changsha, Hunan, China; b Hunan Key Laboratory of Ophthalmology, Central South University, Changsha, China; c National Clinical Research Center for Geriatric Disorders, Xiangya Hospital, Central South University, Changsha, China; d National Clinical Key Specialty of Ophthalmology, Changsha, Hunan, China.

**Keywords:** Alzheimer’s disease, brain cortical structure, glaucoma, IOP, Mendelian randomization, visual field defects

## Abstract

Glaucoma is a heterogeneous group of diseases which is one of the leading causes of irreversible blindness worldwide. Although the eye-brain axis has been proposed, its functional connectivity remains poorly defined. This study aimed to explore the mechanisms and causal relationship between glaucoma and brain cortical structure, focusing on the eye-brain axis. A Mendelian randomization (MR) study was conducted using inverse variance weighting as the primary estimator, alongside MR-PRESSO, MR-Egger, and weighted median methods to assess sensitivity, heterogeneity, and pleiotropy. Pathway analysis, transcriptomic analysis, and weighted gene co-expression network analysis (WGCNA) were applied to investigate brain-eye interactions in Alzheimer’s disease (AD) and primary open-angle glaucoma (POAG), revealing shared pathogenic mechanisms. Significant associations between glaucoma and brain cortex regions, including the superior temporal sulcus, anterior cingulate, cuneus, entorhinal, inferior temporal, and insula, were identified. About 18 overlapping genes between AD and POAG were found, including MYH14, EFNA1, FZD1, and CACNG3. Using WGCNA, 11 overlapping genes were identified as most related to both AD and POAG, including TSC2, MAGED4, LSS, and DNM1. These results contributed to understanding the association between glaucoma and the brain, indicating the eye-brain axis and may provide clues for early screening of high-risk populations.

## 1. Introduction

Glaucoma is a heterogeneous group of diseases characterized by cupping of the optic nerve head and visual field damage, potentially leading to irreversible blindness.^[1–3]^ A study using UN World Population Prospects data estimated that by 2040, 3.54% of affected people will be 40 to 80 years old and 111.8 million will be affected overall.^[4]^ Glaucoma shares similar pathogenic mechanisms with several neurodegenerative and psychiatric diseases, such as Alzheimer’s disease (AD) and depression.^[5]^ This suggested a potential correlation between glaucoma and structural changes in the brain.

**Figure 1.**
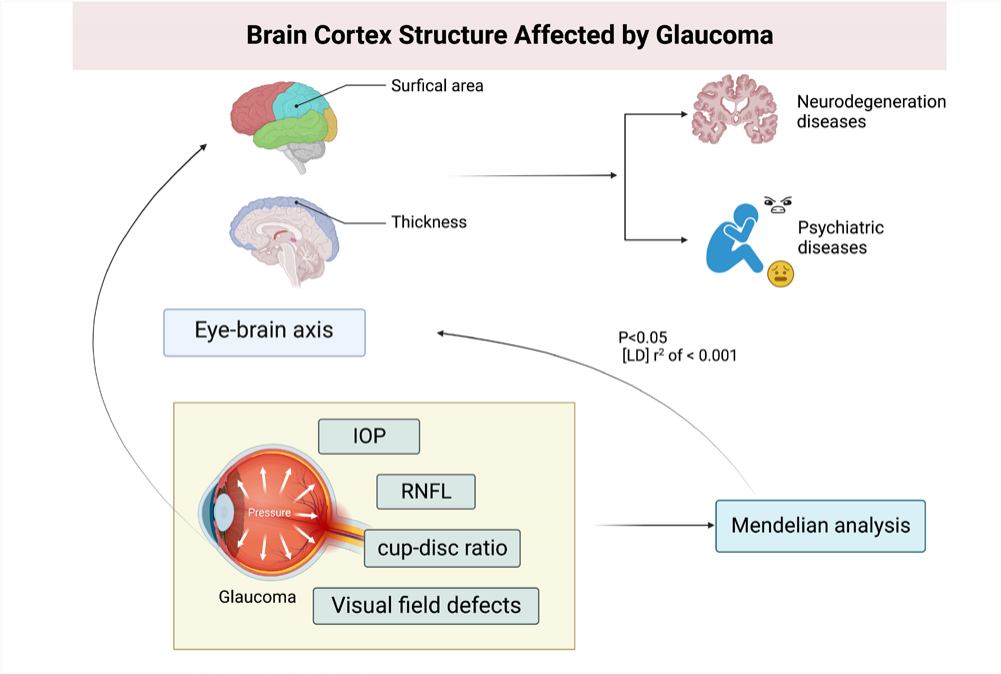
The conception atlas of eye-brain axis. Description of the concept construction of eye-brain axis. IOP = intraocular pressure, RNFL = retinal nerve fiber layer.

The concept of the eye-brain axis has recently emerged, emphasizing the necessity of an intact functional connection between the retina and brain for proper visual function.^[6]^ Functional magnetic resonance imaging provides a noninvasive method to evaluate neuronal activity, revealing neuronal degeneration in the lateral geniculate nucleus (LGN), primary visual cortex (V1), and other visual areas in the context of visual defects, optic disc damage, and retinal nerve fiber layer (RNFL) thinning.^[6–8]^ Additionally, recent research has established a correlation between glaucoma and white matter abnormalities.^[9]^ Previous studies have shown that amyloid-β (Aβ) was involved in the loss of RGCs, as well as the degeneration of the LGN and V1 in glaucoma models. The deposition of Aβ was broadly discovered in the chronic glaucoma model.^[10]^ Therefore, a comprehensive understanding of the eye-brain axis could lead to more advanced treatments for patients (Fig. 1).

We conducted Mendelian randomization (MR) analysis to explore the causal relationship between glaucoma and brain function, specifically focusing on the human brain’s cortical surface area (SA) and cortical thickness (TH). We further discussed the similarities and differences between AD and glaucoma, uncovering the correlation between the 2 diseases. Our research addressed a critical gap, offering new insights into the connection between glaucoma and brain cortex structure, which could enhance early diagnosis and detection of both conditions.

## 2. Methods

### 2.1. Study overview

**Figure 2.**
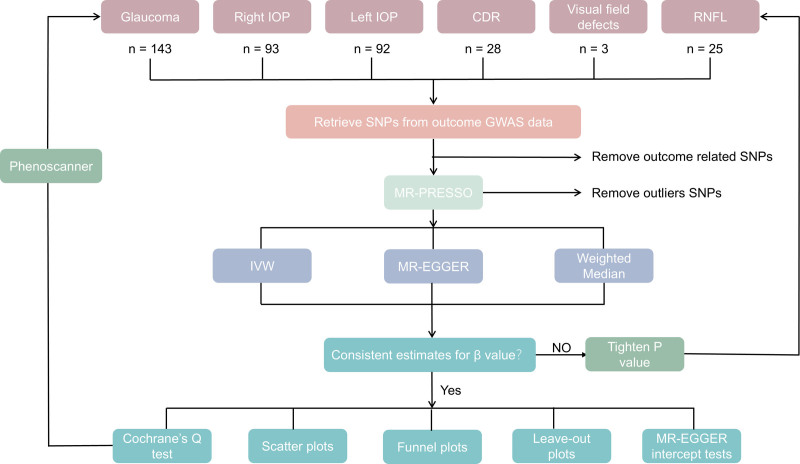
Overview of Mendelian randomization study design. A flow diagram of our MR study design to study the causal relationship of glaucoma on the SA and TH of total and 34 regional brain cortexes. The MR analysis was primarily estimated using inverse variance weighted (IVW). CDR = cup-disc ratio, MR‑Egger and weighted median. GWAS = genome-wide association study, IOP = intraocular pressure, IVW = inverse variance weighted, MR = Mendelian randomization, RNFL = retinal nerve fiber layer, SA = surface area, SNPs = single nucleotide polymorphisms, TH = thickness.

We used a 2-sample MR study to investigate the causal relationship of glaucoma including glaucoma, intraocular pressure (IOP), vertical optic cup-disc ratio (CDR), visual field defects, and RNFL thickness on brain functional cortex. An overview of the study is presented in Figure 2. Glaucoma, IOP, CDR, visual field defects, and RNFL thickness were the exposures, while SA and TH of specific brain regions were the outcomes in the MR analysis. The overall design of this MR study was based on the below assumption: all the included genetic variants were closely related to the interest exposures (glaucoma and its related vision indicators); the genetic variants were not correlated with potential confounders; and the affected effect on the outcome (brain regional cortex) was the only the result of our exposures.

### 2.2. Data source of glaucoma, IOP, CDR, visual field defects, RNFL thickness

The genome-wide association study (GWAS) data associated with glaucoma were extracted from UK Biobank (UKB) participants (n = 3,51,696).^[11]^ The GWAS data associated with left or right eye of IOP (Goldmann-correlated) were extracted from UKB participants. The meta-analysis data of CDR gene loci included 18 cohorts of the European descent (n = 32,067, enrolled cohorts were listed in Table S1, Supplemental Digital Content, https://links.lww.com/MD/P928).^[12]^ The visual field defects data was extracted from the results of FINGEN (n = 3,77,277, https://www.finngen.fi/fi).^[13]^ The FinnGen study combined the genome information with the digital healthcare data of participants whose were over 18 years lived in Finland. The meta-analysis data of RNFL was derived from the optical coherence tomography images of 31,434 UKB participants.^[14]^

### 2.3. Data source of brain cortex SA and cortex TH

The meta-analysis data of brain cortex SA and TH was obtained from ENIGMA Consortium.^[15]^ The study identified cortical SA and TH measures in 51,665 individuals from 60 cohorts all over the world primarily consisting of ~94% European descent (enrolled cohorts were listed in Table S2, Supplemental Digital Content, https://links.lww.com/MD/P928). The brain cortex was divided into 34 regions according to Desikan–Killiany atlas.^[15]^ The boundaries of region were defined by gyral anatomy labeled from the depths of the sulci.^[16]^ The data used here included total SA and average TH, as well as regional SA and TH averaged across both hemispheres.^[15]^ We performed MR analysis for glaucoma, IOP (including the right and the left IOP), CDR, visual field defects, retinal nerve fiber layer (RNFL) thickness on the total SA and TH, and the respective regional SA and TH for 34 regions with or without global weighted. The MR analysis totally generated 828 outcomes.

### 2.4. Genetic variants selection criteria

To identify the causal relationship between glaucoma and the brain cortical function, we used 6 sets of genetic instruments indicating different aspects of glaucoma pathophysiology, including index single nucleotide polymorphisms (SNPs) representing glaucoma (listed in Table S3, Supplemental Digital Content, https://links.lww.com/MD/P928), index SNPs representing right IOP (RIOP, listed in Table S4, Supplemental Digital Content, https://links.lww.com/MD/P928), index SNPs representing left IOP (LIOP, listed in Table S5, Supplemental Digital Content, https://links.lww.com/MD/P928), index SNPs representing CDR (listed in Table S6, Supplemental Digital Content, https://links.lww.com/MD/P928), index SNPs representing visual field defects (listed in Table S7, Supplemental Digital Content, https://links.lww.com/MD/P928), and index SNPs representing RNFL thickness (listed in Table S8, Supplemental Digital Content, https://links.lww.com/MD/P928). Since the diagnosis of glaucoma can’t be accomplished by using a single index, we included a series of index in our MR to fix the bias.

We used stringent clumping criteria (GWAS-correlated *P*-value of 5 × 10^−8^, a linkage disequilibrium [LD] *r*^2^ of <0.001, and <10,000 kb from the index variant) to select independent SNPs.^[17]^ The exception of GWAS-correlated *P*-value for visual defects was 5 × 10^−6^, as applying a more stringent threshold of *P* < 5 × 10^−8^ resulted in too few available instrumental variables. This threshold was chosen to ensure sufficient statistical power while capturing a broader range of genetic variants potentially relevant to visual defects. And we harmonized the SNPs of exposures to exclude palindromic and incompatible SNPs. The concrete selection process is listed below as Figure 2.

### 2.5. Statistics analysis

All analyses were performed using the TwoSampleMR package (version 0.5.6) in R software (version 4.2.2).^[18]^ For a global-level test, a significant 2-sided *P*-value was set as .05. For region-level analyses, given the 816 MR estimates, a Bonferroni-corrected *P*-value was set as 0.05/816 (6.127 × 10^−5^), and meanwhile *P* < .05 was regarded as nominally significant.

We used random-effect inverse variance weighted (IVW),^[19]^ MR-Egger,^[20]^ and weighted median^[21]^ to conduct MR analysis which guaranteed the accuracy and credibility of these results. The primary analysis was conducted using IVW, which relies on inverse variance weighting. This approach incorporated SNP-specific estimates derived from Wald ratios, under the assumption that no directional pleiotropic effects were present for any SNP. MR-Egger and weighted median were used as sensitivity analyses to assess the robustness of IVW. MR-Egger intercept test was utilized to assess the horizontal pleiotropy. No significant evidence of horizontal pleiotropy was detected as indicated by the MR-Egger intercept. Additionally, a leave-one-out analysis was conducted by systematically excluding 1 SNP at a time and applying the IVW method to the remaining SNPs to assess the influence of individual variants on the estimates. If the results of IVW, MR-Egger and weighted median were not consistent, a tighten instrument *P*-value threshold was used and then the MR analysis was re-performed (Table S9, Supplemental Digital Content, https://links.lww.com/MD/P928).^[22]^

We also used the MR-PRESSO outlier test to remove the outlier results and calculated *P*-values to assess pleiotropy for each SNP, while the MR-PRESSO global test evaluated overall horizontal pleiotropy.^[23,24]^ In addition, the Cochran’*Q* test was used to exam the heterogeneity. The funnel plot was used to test directional pleiotropy. After retrieving significant results, we also checked whether the SNPs were associated with potential risk factors on the website the PhenoScanner including hypertension, hyperlipemia, body mass index, drinking, obesity, smoking, and neuropsychiatric diseases, and removed the related SNPs, after which we conducted the MR analysis again to confirm the accuracy of results.^[25]^

### 2.6. Integration of transcriptomic analysis in the GEO database

Data for POAG and AD were extracted from Gene Expression Omnibus (GEO) microarray expression profiling dataset (http://www.ncbi.nlm.nih.gov/geo/). We chose dataset including GSE27276 and GSE260873. Dataset GSE27276 involved genome-wide expression in human trabecular meshwork tissue between 13 controls and 15 POAG cases. Dataset GSE260873 utilized in this study involved RNA sequencing data isolated from the gray matter in the superior frontal gyrus from 12 patients with AD and 12 individuals without AD. The data in GSE260873 and GSE27276 have been normalized when uploaded in GEO datasets. Specifically, for GSE260873, we applied a log_2_ transformation to the normalized expression values (log_2_(normalized expression + 1)). For GSE27276, the expression levels have already been normalized and log_2_-transformed, making the data ready for downstream analysis without the need for further preprocessing. For batch correction, we applied the surrogate variable analysis to both datasets. The differential expressed genes (DEGs) respectively related to POAG and AD were screened by the “limma” package in R software. The genes with *P*-value < .05 and |log_2_(FC)| > 0.5 were considered to be DEGs. Then, the “ggplot2” packages in R software are used to draw heatmap and volcano plot respectively to visualize the differential genes. The venn plot of overlapping protein between POAG and suicide/AD were pictured by using “ggvenn” package. Gene Ontology (GO) terms and Kyoto Encyclopedia of Genes and Genomes (KEGG) pathways enrichment analysis were performed to find the functional enrichment of the above overlapping mRNA.

### 2.7. WGCNA analysis

Weighted correlation network analysis (WGCNA) was used in this study to discovering clusters (modules) of highly correlated genes and summarize such clusters using the module eigengene.^[26]^ For GSE27276 and GSE260873, we used WGCNA analysis and find the most related modules and key genes. We chose *R*^2^ = 0.9 and the soft-threshold β = 18. Subsequently, the adjacency matrix was transformed into a topological overlap matrix (TOM). Modules were identified with hierarchical clustering (minModuleSize = 50). The eigengene was calculated, and the modules were hierarchically clustered. The module eigengene (ME) was used to distinguish the vital modules associated with POAG or AD. ME shows the first principal component in the module, and describes the expression pattern of the module. GO terms and KEGG pathways enrichment analysis were performed to find the functional enrichment of the gene module above.

### 2.8. Ethical review

Since all data used in this study were obtained from publicly available databases, ethical approval and patient consent were not required. The data for this study was sourced from the the UKB, FinnGen, ENIGMA and GEO public database and can be extracted respectively from https://www.nealelab.is/uk-biobank, https://www.finngen.fi/fi, https://enigma.ini.usc.edu/ and http://www.ncbi.nlm.nih.gov/geo/.

## 3. Results

### 3.1. Causal effects of glaucoma on brain cortex

**Figure 3.**
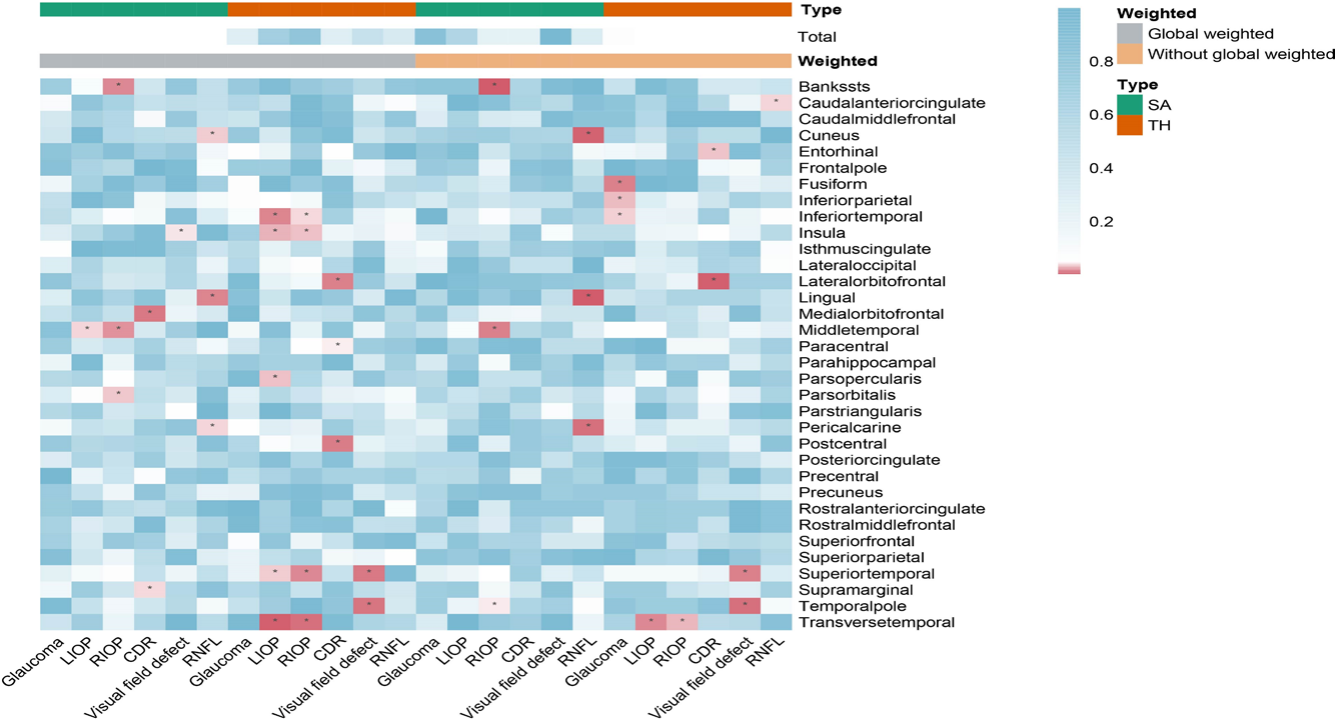
Heatmap for the IVW estimates of glaucoma and brain cortical structure. IVW estimates from glaucoma, right intraocular pressure, left intraocular pressure, CDR, visual field defects and retinal nerve fiber layer on brain cortical structure as defined using magnetic resonance imaging-measured brain cortical surficial area and thickness. The color of each block represents the IVW-derived *P*-values in which *P*-values of <.05 were shown in red and *P*-values of >.05 were shown in white or blue. *P*-value < .05 is set as nominally significant, whereas <6.127 × 10^−5^ is set as significant. CDR = cup-disc ratio, IVW = inverse variance weighted, LIOP = left intraocular pressure, RIOP = right intraocular pressure, RNFL = retinal nerve fiber layer, SA = surface area, TH = thickness.

A total of 143 index SNPs were selected to genetically predict glaucoma, while 92 and 93 index SNPs were chosen to indicate left and right IOP, respectively. Additionally, 25 index SNPs were selected for RNFL thickness, 28 for CDR, and 3 for visual defects. Notably, 34 SNPs overlapped between left and right IOP, which was acceptable as they were representative index SNPs for IOP. Consequently, we conducted MR analysis between 6 representative aspects of glaucoma and 34 regional cortical SA and TH measurements, both with and without global weighting (Fig. 3). Furthermore, we analyzed the causal relationship between the glaucoma and brain cortex SA and TH (Fig. 3). The IVW results were the primary components of our analysis. No significant glaucoma-associated SNPs were found to influence the global cortical level. However, we identified several nominally significant SNPs that may affect specific functional regions of the gyrus in relation to glaucoma (Fig. 3). Glaucoma index SNPs had no causal effect on the regional brain cortex SA with or without global weighted. Data indicated that glaucoma function had a potential influence on the SA and TH region including gyrus of bankssts, caudal anterior cingulate, cuneus, entorhinal, fusiform, inferior parietal, inferior temporal, insula, lateral orbitofrontal, lingual, medial orbitofrontal, middle temporal, paracentral, pars opercularis, pars orbitalis, pericalcarine, postcentral, superior temporal, supramarginal, temporal pole, transverse temporal. Detailed information is provided in Table 1 and the supplementary tables. We further created scatter plots, funnel plots, and leave-out plots for all genetically predicted SNPs for 6 aspects of glaucoma and the potentially affected SA and TH with or without global weighted (Figs. S1–S18, Supplemental Digital Content, https://links.lww.com/MD/P927). For all nominally significant SNPs, we used the Phenoscanner website to exclude those influenced by risk factors (as listed in Table S10, Supplemental Digital Content, https://links.lww.com/MD/P928). The MR-Egger intercept test *P*-values were all >.05 for IVW significant SNPs, indicating no horizontal pleiotropy.

**Table 1:** Nominally significant Mendelian randomization estimates from glaucoma, RIOP, LIOP, CD ratio, visual defects, RNFL on genetically predicted cortical structure.

Exposure	Regions	Outcome	IVW-derived *P*-value	SE	β (95% confidence intervals)	Cochran’s *Q*-derived *P*-value (IVW)	MR-Egger intercept-derived *P*-value
RIOP	SA with global weighted	Bankssts	.01688415	3.153644	−7.534751 (−13.71589324 to −1.35360876)	.1610958	.789299
RNFL	SA with global weighted	Cuneus	.03577377	0.9637074	0.03577377 (1.853092734–1.924640274)	.3953804	.8227772
Visual field defect	SA with global weighted	Insula	.04248628	5.820073	11.80733 (0.39999–23.21467)	.885334	.7933541
RNFL	SA with global weighted	Lingual	.01522818	2.28831	5.553515 (1.068–10.0386)	.07561273	.3415702
CDR	SA with global weighted	Medial orbitofrontal	.01158722	6.282351	−15.85962 (−28.1730 to −3.5462)	.4161612	.06997356
LIOP	SA with global weighted	Middle temporal	.03853515	7.749717	−16.035069 (−31.2245 to −0.8456)	.004543756	.5970609
RIOP	SA with global weighted	Middle temporal	.01902528	7.288442	−17.09165 (−37.3770 to −2.8064)	.04548642	.7422792
RIOP	SA with global weighted	Pars orbitalis	.03452194	1.61511	3.414216 (0.2486–6.5798)	.8465002	.8388516
RNFL	SA with global weighted	Pericalcarine	.03788488	1.423034	2.954358 (0.1652–5.7435)	.1622568	.6868197
CDR	SA with global weighted	Supramarginal	.03867818	14.80571	30.6122 (1.5930–59.6314)	.5295423	.9152001
LIOP	TH with global weighted	Inferior temporal	.01585135	0.003035155	0.007321765 (1.372E−3–0.0133)	.482825	.6329547
RIOP	TH with global weighted	Inferior temporal	.03885252	0.003152263	0.006511773 (3.33E−04–1.27E−02)	.3137848	.161031
LIOP	TH with global weighted	Insula	.02825338	0.003590151	0.007875894 (8.391E−04–1.49E−02)	.04422644	.4502293
RIOP	TH with global weighted	Insula	.032344755	0.003395193	0.007266134 (6.12E−04–1.39E−02)	.1659844	.3000331
CDR	TH with global weighted	Lateral orbitofrontal	.01427768	0.005478756	0.01342405 (2.69E−03–2.42E−02)	.2847521	.2742373
CDR	TH with global weighted	Paracentral	.04550759	0.004907963	0.009815593 (1.96E−04–1.94E−02)	.3908524	.2844665
LIOP	TH with global weighted	Pars opercularis	.03217115	0.002545815	−0.005453843 (−1.04E−02 to −4.64E−04)	.279893	.4653224
CDR	TH with global weighted	Postcentral	.01336995	0.004271543	−0.01056677 (−1.89E−02 to −2.19E−3)	.3777455	.5570059
LIOP	TH with global weighted	Superior temporal	.03562407	0.003011846	−0.006328468 (−1.22E−02 to −4.25E−04)	.2280699	.9167432
RIOP	TH with global weighted	Superior temporal	.01696313	0.003398077	−0.008112929 (−1.48E−02 to −1.45E−03)	.01128277	.2270669
Visual field defect	TH with global weighted	Superior temporal	.01183891	0.003803505	−0.00957308 (−1.70E−02 to −2.12E−03)	.3329308	.4519864
Visual field defect	TH with global weighted	Temporal pole	.01089616	0.009326843	−0.023746252 (−4.20E−02 to −5.47E−03)	.6769357	.8193353
LIOP	TH with global weighted	Transverse temporal	.00420875	0.004599944	−0.01316539 (−2.22E−02 to −4.15E−03)	.4847958	.1118366
RIOP	TH with global weighted	Transverse temporal	.00905521	0.004880191	−0.01273712 (−2.23E−02 to −3.17−03)	.2405437	.3972771
RIOP	SA without global weighted	Bankssts	.003989793	3.966734	−11.4200986 (−19.2 to −3.65)	.1100141	.3803361
RNFL	TH without global weighted	Caudal anterior cingulate	.0382417	0.001339957	−0.002776732 (−0.00540304772 to −0.00015041628)	.552215	.2748972
RNFL	SA without global weighted	Cuneus	.00629431	1.209682	3.304901 (0.93392428–5.67587772)	.4015765	.516554
CDR	TH without global weighted	Entorhinal	.03263177	0.01494659	0.03193465 (0.00263933360000001–0.0612299664)	.6248359	.4863026
Glaucoma	TH without global weighted	Fusiform	.01402422	0.001588878	−0.003903305 (−0.00701750588 to −0.00078910412)	.3124801	.1755189
Glaucoma	TH without global weighted	Inferior parietal	.03089072	0.001408483	−0.003040179 (−0.00580080568 to −0.00027955232)	.9090512	.9706877
Glaucoma	TH without global weighted	Inferior temporal	.03629676	0.001669538	−0.003495323 (−0.00676761748 to −0.00022302852)	.8403206	.5607643
CDR	TH without global weighted	Lateral orbitofrontal	.005211349	0.006671737	0.01863864 (0.00556203548–0.03171524452)	.7788618	.2786813
RNFL	TH without global weighted	Lingual	.002939873	2.774716	8.251884 (2.81344064–13.69032736)	.10954548	.7468527
RIOP	SA without global weighted	Middle temporal	.01372171	12.0184	−29.61896 (−53.175024 to −6.062896)	.009174386	.1796473
CDR	TH without global weighted	Paracentral	.140958	0.007239849	0.01559431 (0.00140420596–0.02978441404)	.3719876	.7373556
RNFL	SA without global weighted	Pericalcarine	.009817014	1.64111	4.237689 (1.0211134–7.4542646)	.1488886	.7752245
RIOP	SA without global weighted	Temporal pole	.04377713	1.710582	−3.4489069 (−6.80164762 to −0.09616618)	.001727006	.5758234
Visual field defect	TH without global weighted	Temporal pole	.009320226	0.01030391	−0.02679099 (−0.0469866536 to −0.0065953264)	.5633278	5.59E-01
LIOP	TH without global weighted	Transverse temporal	.01610277	0.006087239	−0.01464941 (−0.02658039844 to −0.00271842156)	.07879453	5.19E-01
RIOP	TH without global weighted	Transverse temporal	.03021788	0.005888416	−0.01276151 (−0.02430280536 to −0.00122021464)	.1714643	.6457767

CDR = cup-disc ratio, IVW = inverse variance weighted, LIOP = left intraocular pressure, MR = Mendelian randomization, RIOP = right intraocular pressure, RNFL = retinal nerve fiber layer, SA = surface area, TH = thickness.

### 3.2. Transcriptomic analyses in GEO database

**Figure 4.**
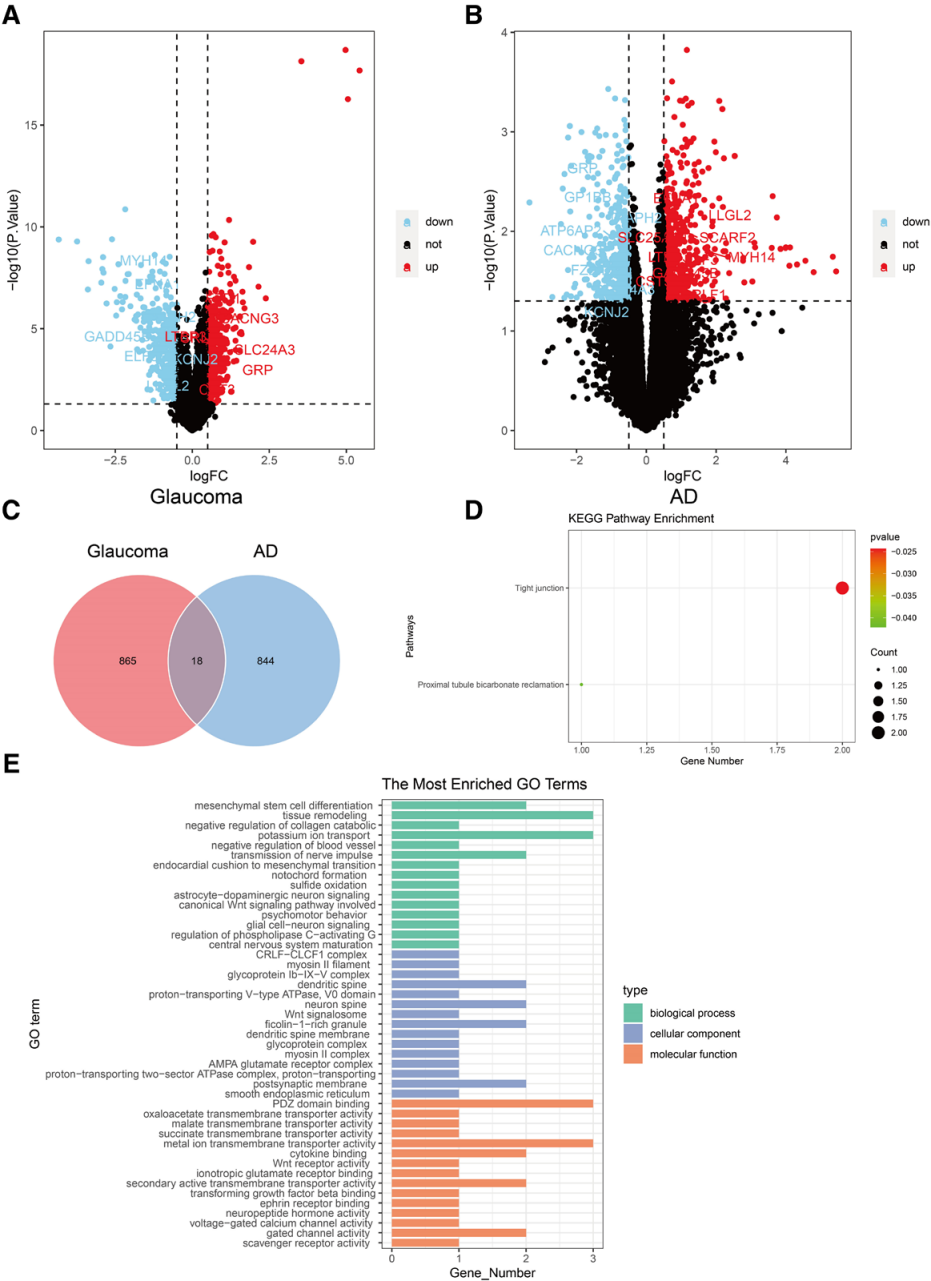
Integration of transactional analysis of AD and POAG. (A) Volcano plot for DEGs in POAG. (B) Volcano plot for DEGs in AD. (C) Venn diagram summarizing the differential and overlapping DEGs for POAG and AD. (D) KEGG enrichment analysis for overlapping DEGs. (E) GO analysis for overlapping DEGs. AD = Alzheimer’s disease, DEGs = differentially expressed genes, KEGG = Kyoto Encyclopedia of Genes and Genomes, GO = Gene Ontology, POAG = primary open-angle glaucoma.

To further testify the causal relationship of glaucoma on brain cortex, we compared DEGs for the data in GEO database for primary open-angle glaucoma (POAG) and AD. This study aimed to reveal the similarities and differences of these 2 diseases and find the close relationship between POAG and AD from the disease level. In particular, the data of patients with AD were extracted from inferior frontal which was found in the previous study to be related with glaucoma. In GSE27276 dataset for POAG, we found 444 upregulated genes and 439 downregulated genes (Fig. 4A, Table S11, Supplemental Digital Content, https://links.lww.com/MD/P928). In GSE260873 dataset for AD, we found 509 up-regulated genes and 352 down-regulated genes (Fig. 4B, Table S12, Supplemental Digital Content, https://links.lww.com/MD/P928). We identified 18 overlapping mRNA between POAG and AD including MYH14, EFNA1, FZD1, CACNG3, LTBP3, DIAPH2, GADD45B, ELF3, CRLF1, KCNJ2, SLC24A3, GP1BB, GRP, SLC25A10, ATP6AP2, SCARF2, LLGL2, and CST3 (Fig. 4C). Then we conducted KEGG enrichment analysis and GO enrichment analysis to find the common influenced pathway. We found these DEGs involved in tight junction and proximal tubule bicarbonate reclamation (Fig. 4D). From the GO enrichment analysis, the crucial common involved in the common pathological process can be identified (Fig. 4E).

### 3.3. Module hub gene analysis by WGCNA

**Figure 5.**
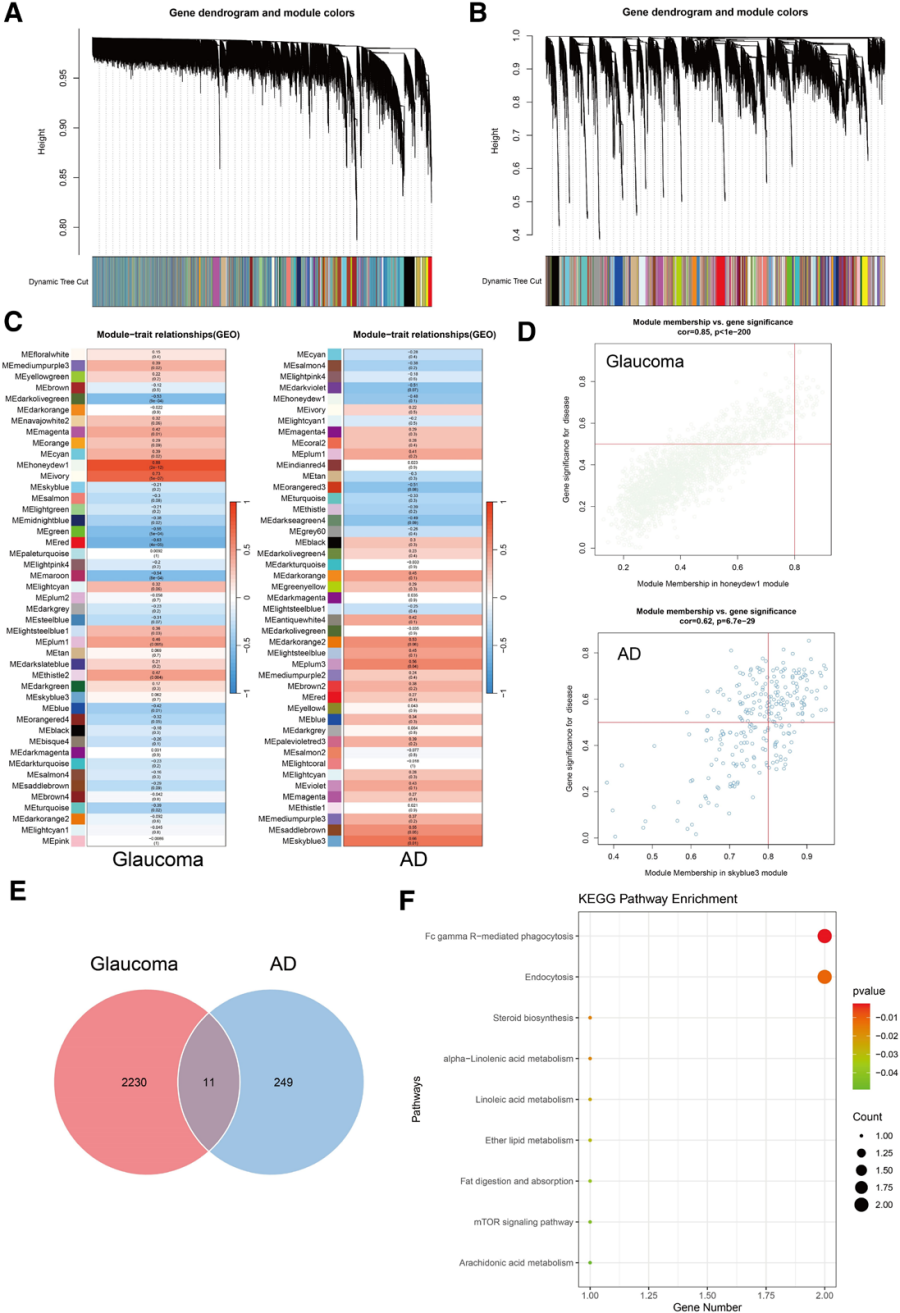
WGCNA analysis for GSE27276, and GSE260873. (A) Dynamic tree plot for glaucoma. (B) Dynamic tree plot for AD. (C) Specific and concrete gene module division and correlation index respectively for glaucoma and AD. (D) Significance and correlation index for genes in the most related gene modules in POAG and AD. (E) Venn diagram summarizing the differential and overlapping genes between the most related gene module for POAG and AD. (F) KEGG analysis for overlapping genes in the most related gene modules. POAG = primary open-angle glaucoma, AD = Alzheimer’s disease, KEGG = Kyoto Encyclopedia of Genes and Genomes, WGCNA = weighted gene co-expression network analysis.

We used WGCNA analysis to establish gene modules which contained a group of high expression genes (Fig. 5A, B). For the GSE27276 dataset, 45 gene modules were identified (Fig. 5C). Among these, module MEhoneydew1 was found to be the most correlated with glaucoma development (Fig. 5D, Table S13, Supplemental Digital Content, https://links.lww.com/MD/P928). Also, for GSE260873, 45 gene modules were identified (Fig. 5C), among which MEskyblue3 was considered to be the most intimate module for AD development (Fig. 5D, Table S13, Supplemental Digital Content, https://links.lww.com/MD/P928). By combining the 2 modules, we identified 11 overlapping genes including TSC2, MAGED4, LSS, MIDN, EPS15, DNM1, GAK, LGALS3BP, PLA2G6, TUBG2, and MAEA (Fig. 5E). Later, we performed KEGG analysis at the RNA level (Fig. 5F). We identified several signal pathways involved in both AD and glaucoma pathology. They included Fc gamma R-mediated phagocytosis, endocytosis, lipid metabolism (steroid biosynthesis, alpha-linolenic acid metabolism, linoleic acid metabolism, ether lipid metabolism, fat digestion and absorption and arachidonic acid metabolism) and mammalian target of rapamycin (mTOR) signaling pathway (Table S14, Supplemental Digital Content, https://links.lww.com/MD/P928).

## 4. Discussion

To date, this was the first large-scale MR analysis focusing on the relationship between glaucoma function and the brain functional structure. Our findings indicated that glaucoma, IOP, RNFL thickness, CDR, and visual field defects impacted brain function, further supporting the existence of the eye-brain axis. This discovery explained the reason why glaucoma patients often exhibited a higher prevalence of brain structure changes and brain diseases. These results had significant implications for diagnosing and preventing brain diseases and offered valuable insights for clinical practice.

RGCs degeneration is one of the main characters for glaucoma. Previous study has demonstrated that multiple forms of death were involved in the RGCs degeneration including apoptosis, ferroptosis, PANoptosis, and necrosis.^[27–32]^ We mainly demonstrated that several brain cortical regions including visual and nonvisual ways where SA and TH were affected by glaucoma. On the one hand, the mechanism of transneuronal degeneration can be a contributing factor to defects in the visual pathway. Anterograde transsynaptic degeneration is the process in which retinal degeneration causes subsequent degeneration of the posterior visual pathway. The axons of retinal neurons extend to form the optic nerve and converge at the optic chiasm, then project to the visual cortex through the LGN.^[33]^ Thus, transneuronal degeneration may be one of the causes of cortical changes in the brain. Glaucoma involves transneuronal degeneration of the posterior structures along the central visual pathway, as the neurotrophic factor like brain-derived neurotrophic factor (BDNF) and ciliary neurotrophic factor (CTNF), and metabolites like choline plays a significant part in it.^[34]^ The volume of primary visual cortex and visual pathway including LGN, V1, lingual gyrus and calcarine fissure decreases indicating the atrophy of these structures. Meanwhile, the degeneration of brain cortex along the visual pathway was discovered in POAG including hippocampus, thalamus and midbrain as the magnetic resonance imaging indicating decreased volume.^[35]^ On the other hand, nonvisual cognitive pathways were also influenced by POAG like cingulate cortex, caudate nucleus, corpus callosum and claustrum.^[35]^ This might explain the emotion change in glaucoma patients.^[36]^ Intrinsically photosensitive retinal ganglion cells detect light via the G-protein-coupled receptor melanopsin, depolarize, generate electrical spikes, and influence physiology, behavior, perception, and mood through widespread brain projections to cortex.^[37]^This will be the physiological basis for brain cortex changes.

Among these, most of the temporal cortical regions, including the superior, middle, transverse, and inferior temporal gyri, fusiform gyrus, and temporal pole, were found to be affected by glaucoma-related SNPs. Observation of Tau protein in brain can be the preclinical characteristics of AD which widespread deposits in these area.^[38]^ Our results proved the influence of glaucoma on the temporal part on the genetic level which potentially explains the phenomena and outcomes of high incident rates of AD in the population of glaucoma patients. The impact of glaucoma risk SNPs on the insula further elucidated the cognitive dysfunction and memory loss observed in glaucoma patients. Specifically, the pars opercularis (Brodmann area 44), which was involved in language function, was affected. The significant SNPs identified could potentially serve as preclinical markers for brain diseases such as AD. The above results indicated that the nominally significant findings from the MR analysis align with the transneuronal degeneration caused by glaucoma and brain cortical atrophy.

Given the close connection between glaucoma and brain function, our study focused on the relationship and similarities between AD and POAG.^[39,40]^ Previous studies have identified several shared genetic loci and protein-coding genes, such as MTCH2, NDUFS3, SPI1, MYBPC3, and PTPMT1, suggesting common genetic markers between the 2 conditions.^[41]^ In our study, we identified 18 overlapping DEGs between AD and POAG. Among them, GADD45B, FZD1, EFNA1, LTBP3, and CST3 have been reported to potentially play a role in the development of both AD and glaucoma. GADD45B is directly involved in stress-induced DNA repair, cell cycle arrest, cell survival, and apoptosis. It is widely expressed in the nervous system, and its dysregulation may lead to neuronal damage.^[42]^ FZD1 is a receptor in the Wnt signaling pathway, which plays a crucial role in neuronal development, synaptic plasticity, and neurodegeneration. Dysregulation of FZD1 has been linked to neurodegenerative processes in AD.^[43]^ Given its involvement in retinal development, our study suggested that FZD1 dysregulation may contribute to RGCs damage in glaucoma.^[44]^ EFNA1, which is essential for axon guidance and neuronal communication, also plays a key role in synaptic plasticity.^[45]^ The activation of EFNA1 may therefore promote neuronal regeneration, offering potential therapeutic insights for neurodegenerative diseases.^[46]^ LTBP3 regulates the activation of TGF-β, a cytokine involved in tissue repair and fibrosis. In the context of glaucoma, LTBP3 contributes to extracellular matrix remodeling,^[47]^ while mutations in LTBP1 have been identified in clinical families with AD. CST3 encodes cystatin C, a potent inhibitor of cysteine proteases. Elevated levels of CST3 have been associated with modulation of Aβ metabolism in AD.^[47]^ Cysteine, as part of this process, may also be involved in aqueous humor outflow. Consequently, elevated CST3 levels could potentially contribute to glaucoma by increasing intraocular pressure.^[48]^ In addition, these DEGs were found to be enriched in pathways such as tight junctions. The results suggested that alterations in tight junctions may represent a common pathological feature of both diseases. Under stress, downregulation of VE-cadherin, β-catenin, VEGFR-1, VEGFR-2, vimentin, Cdc42, and ACK1 was observed in the retina.^[49]^ In AD mouse model, tight junctions in the intestine can be disrupted by Aβ deposition.^[50]^ For example, Aβ and Tau protein deposits in the retina and central visual systems of experimental high IOP model.^[51]^

To identify the most co-expressed genes in AD and glaucoma, we performed WGCNA analysis. We identified 2 modules highly related to both diseases, showing a positive association with disease progression. By integrating AD and glaucoma data, we discovered 11 overlapping genes. KEGG pathway analysis revealed common functions in lipid metabolism, the mTOR pathway, endocytosis, and phagocytosis. Lipid metabolism plays a complex role in the neurodegenerative changes observed in glaucoma. For example, the leukotriene and arachidonic acid metabolic pathways promote retinal neuroinflammation,^[52]^ which directly leads to RGCs damage. In contrast, the metabolism of lipoxins has been shown to exert a protective effect in glaucoma models. In AD, abnormal lipid levels accelerate Aβ deposition and promote tangled Tau protein.^[53]^ Thus, disruption of lipid metabolism homeostasis may serve as a common mechanism of degeneration in both glaucoma and AD, potentially acting as a bridge in the eye-brain axis. The mTOR signaling pathway plays a crucial role in coordinating various neuronal functions and maintaining neuronal homeostasis in the brain and retina.^[54,55]^ Abnormalities in the mTOR pathway can disrupt autophagic processes, leading to neurotoxic cell death. In AD, glaucoma and other neurodegenerative disorders, misfolded and ubiquitylated proteins accumulate in autophagic vacuoles, clogging the system, and causing the buildup of toxic proteins in dystrophic neurites. Neuronal cells, unlike other cell types, cannot divide to eliminate excess macromolecules or organelles, making them highly dependent on autophagy.^[56,57]^ Any disruption in autolysosome clearance can impair the entire endocytic machinery, which may worsen metabolic and immune-related damage, thereby exacerbating AD and RGCs loss in glaucoma.^[55]^ As noted, damage to endocytosis and phagocytosis can also lead to RGCs loss and neuronal degeneration, contributing to the development of neurodegenerative diseases. What’s more, the transcriptomic analysis presented above reveals that the common pathways identified in both glaucoma and AD may contribute to RGCs loss, and therefore may result in the transneuronal degeneration, and brain cortical atrophy.

All in all, this MR study and transcriptomic analysis revealed common pathways and a close relationship between glaucoma and AD. Existing clinical studies also suggested that glaucoma patients were at a higher risk of developing dementia.^[58]^ Additionally, clinical research indicated that, compared to cataract patients, individuals with normal-tension glaucoma were more likely to experience cognitive impairments.^[58,59]^ These findings aligned with our results and further emphasized the importance of identifying shared mechanisms in neurodegenerative diseases, as well as the need to search for potential preclinical biomarkers.

A limitation of our study was that the databases predominantly consist of European populations, which may not fully represent the genetic and environmental diversity of other populations. This may lead to biased estimates and limit the generalizability of the findings to other ethnic and racial groups. Future studies should aim to include more diverse cohorts to enhance the applicability and robustness of the results across different populations. What’s more, new methods accounting for potential participation biases can be considered in the future.^[60]^ Another potential limitation of our study was the sample overlap between the exposure (glaucoma-related indicators, including glaucoma, IOP, CDR, and RNFL) and outcome (cerebral cortical structure) datasets, both derived from the UKB. Based on Burgess’s simulation,^[61]^ the bias introduced by sample overlap is minimal when the instrument strength is high, with bias tending toward the null. In our study, the total overlap between the CDR cohort and outcome was fewer than 1716 participants, constituting <5% of the sample. Moreover, according to study of Minelli et al,^[62]^ 2-sample MR remains valid even with overlap when both exposure and outcome come from large biobanks like the UKB. Given the large sample size and strong instrument strength in our study, we expected the bias from sample overlap to be limited. As such, no additional sensitivity analysis was deemed necessary. To further ensure the robustness of our findings, we plan to conduct sensitivity analyses, such as MRlap, in future studies.^[63]^ Additionally, previous study employed a similar MR approach using the same outcome as in our research, with exposure data from the UKB, further supporting the validity of this approach.^[64]^ The wide confidence intervals observed for some β coefficients, such as for visual defects and SA of the insula (β = 11.807, 95% CI [0.399, 23.214]), indicate instability in the results. This could be due to factors such as sample size, data variability, or measurement inconsistencies. These uncertainties highlighted the need for caution in interpreting the findings and suggested that further studies with larger sample sizes and more precise measurements are necessary to validate these associations. Additionally, adjusting for *P*-values revealed no significant estimates in the current data and analysis. Furthermore, we relied on public databases to explore the disease pathology between glaucoma and brain diseases. Due to data source constraints and the cross-sectional nature of the study, we were only able to compare AD with glaucoma. As a result, we could not determine the temporal sequence or causal relationship between the 2 conditions. For instance, AD-related cognitive or behavioral symptoms can be examined in commonly used glaucoma animal models, such as the acute glaucoma model,^[27]^ glutamate excitotoxicity model,^[28]^ and chronic magnetic bead-induced model.^[65]^ Based on the insightful feedback provided, we have also emphasized the importance of prospective studies, which will be critical in clarifying the sequence of pathological events and uncovering the molecular basis of glaucoma-related brain alterations.

## 5. Conclusions

This study revealed the relationship between the functional brain cortex and glaucoma, IOP, RNFL, CDR, and visual field defects. We found that glaucoma-related functions might drive broad structural brain changes. Brain magnetic resonance imaging of different functional regions may offer clues for early screening of high-risk populations for psychiatric and neurodegenerative disorders in glaucoma patients. Additionally, we uncovered similarities and differences between POAG and AD, identifying significant and notable potential preclinical markers. Neurologists should consider both retinal and neurodegenerative changes. This study highlighted the potential eye-brain axis and offers valuable clinical insights.

## Acknowledgments

We gratefully thank for the data supplied by GEO database, UK Biobank, FINGEN, and ENIGMA Consortium.

## Author contributions

**Conceptualization:** Xingyi Chen, Xiaobo Xia.

**Investigation:** Chaoran Shi.

**Methodology:** Meihui He, Xingyi Chen.

**Supervision:** Xiaobo Xia.

**Writing – review & editing:** Chaoran Shi.

**Writing – original draft:** Xingyi Chen.

## Supplementary Material


