# Supplementary material for: Novel insights into the relationship between glaucoma and brain diseases from the genetic to diseases levels: A cross-sectional study
Source: Medicine (Baltimore). 2025 Sep 19;104(38):e44416. doi: 10.1097/MD.0000000000044416 (PMC12459570; doi:10.1097/MD.0000000000044416)

Figure S1. Scatter plots of nominally significant estimates from genetically predicted glaucoma on A. without global weighted TH of the fusiform; B. without global weighted TH of the caudal anterior cingulate; C. without global weighted TH of the cuneus.

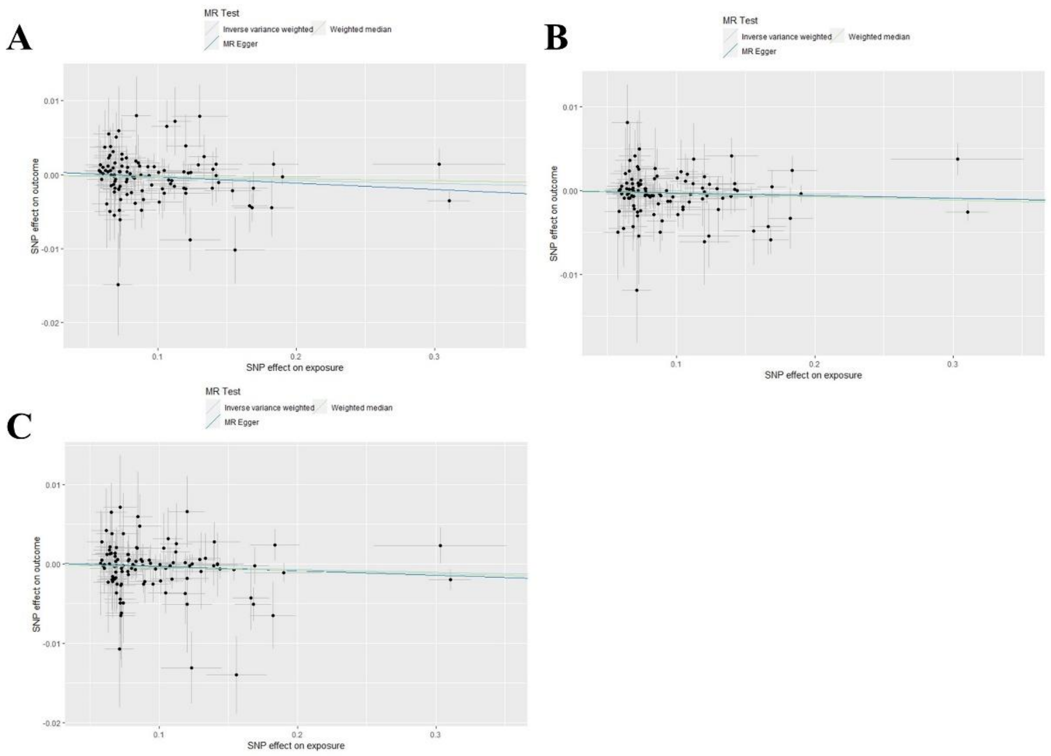

Figure S2. Funnel plots of nominally significant estimates from genetically predicted glaucoma on A. without global weighted TH of the fusiform; B. without global weighted TH of the caudal anterior cingulate; C. without global weighted TH of the cuneus.

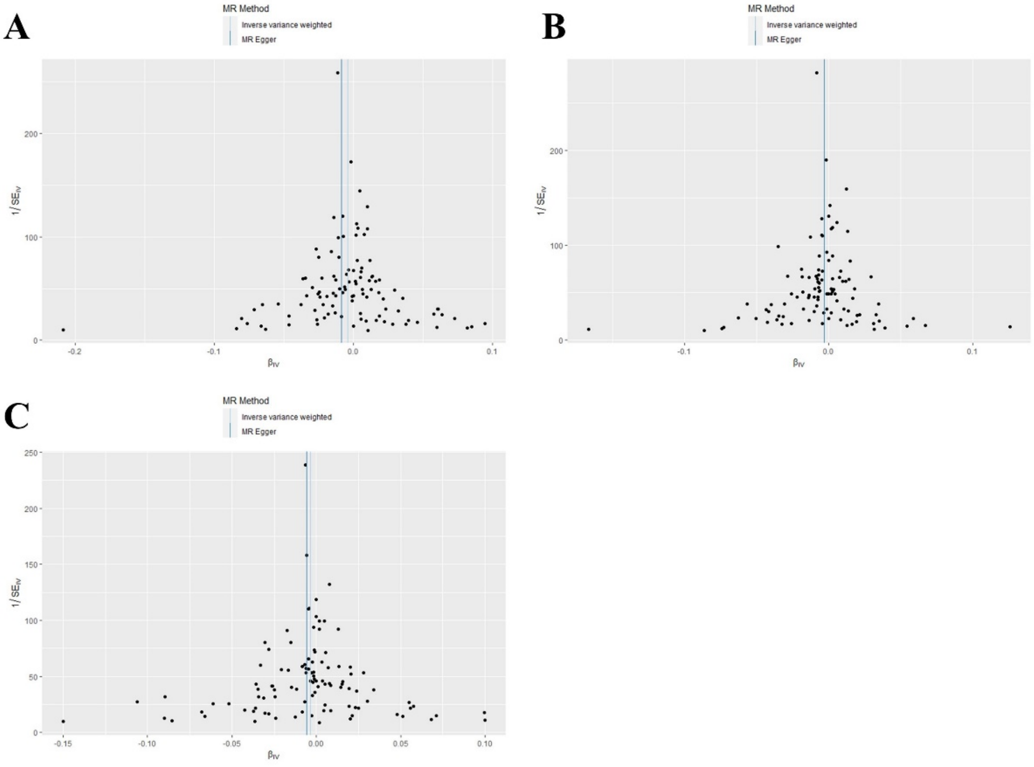

Figure S3. Leave-out plots of nominally significant estimates from genetically predicted glaucoma on A without global weighted TH of the fusiform; B. without global weighted TH of the caudal anterior cingulate; C. without global weighted TH of the cuneus.

A

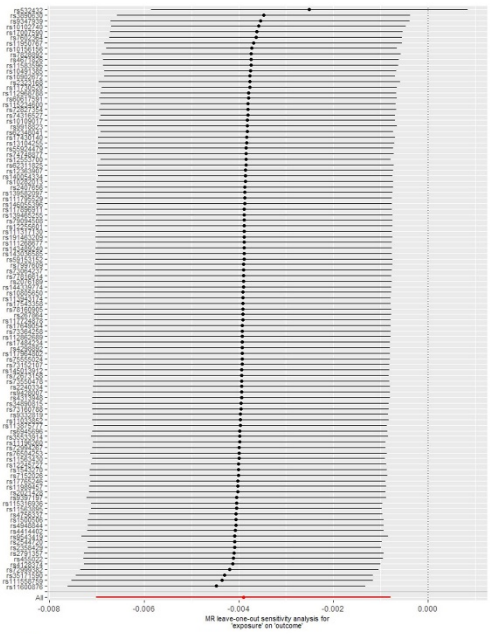

B

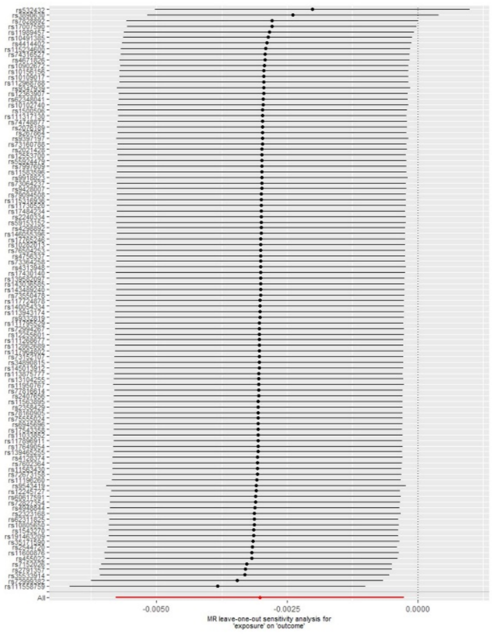

C

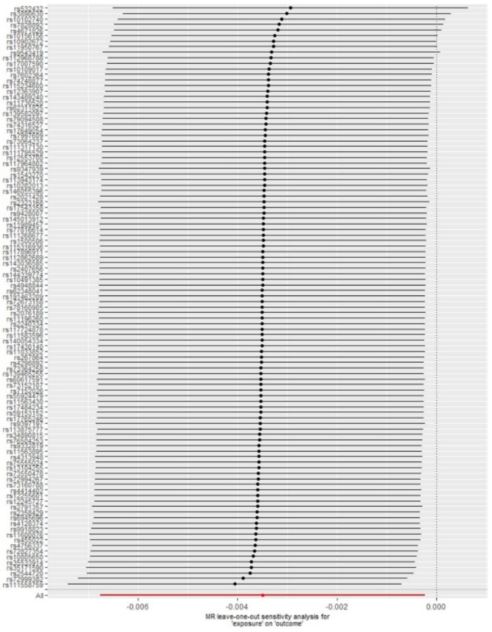

Figure S4. Scatter plots of nominally significant estimates from genetically predicted LIOP on A global weighted SA of the middle temporal; B. global weighted TH of the inferior temporal; C. global weighted TH of the insula; D. global weighted TH of the pars opercularis; E. global weighted TH of the superior temporal; F. global weighted TH of the transverse temporal; G. without global weighted TH of the transverse temporal.

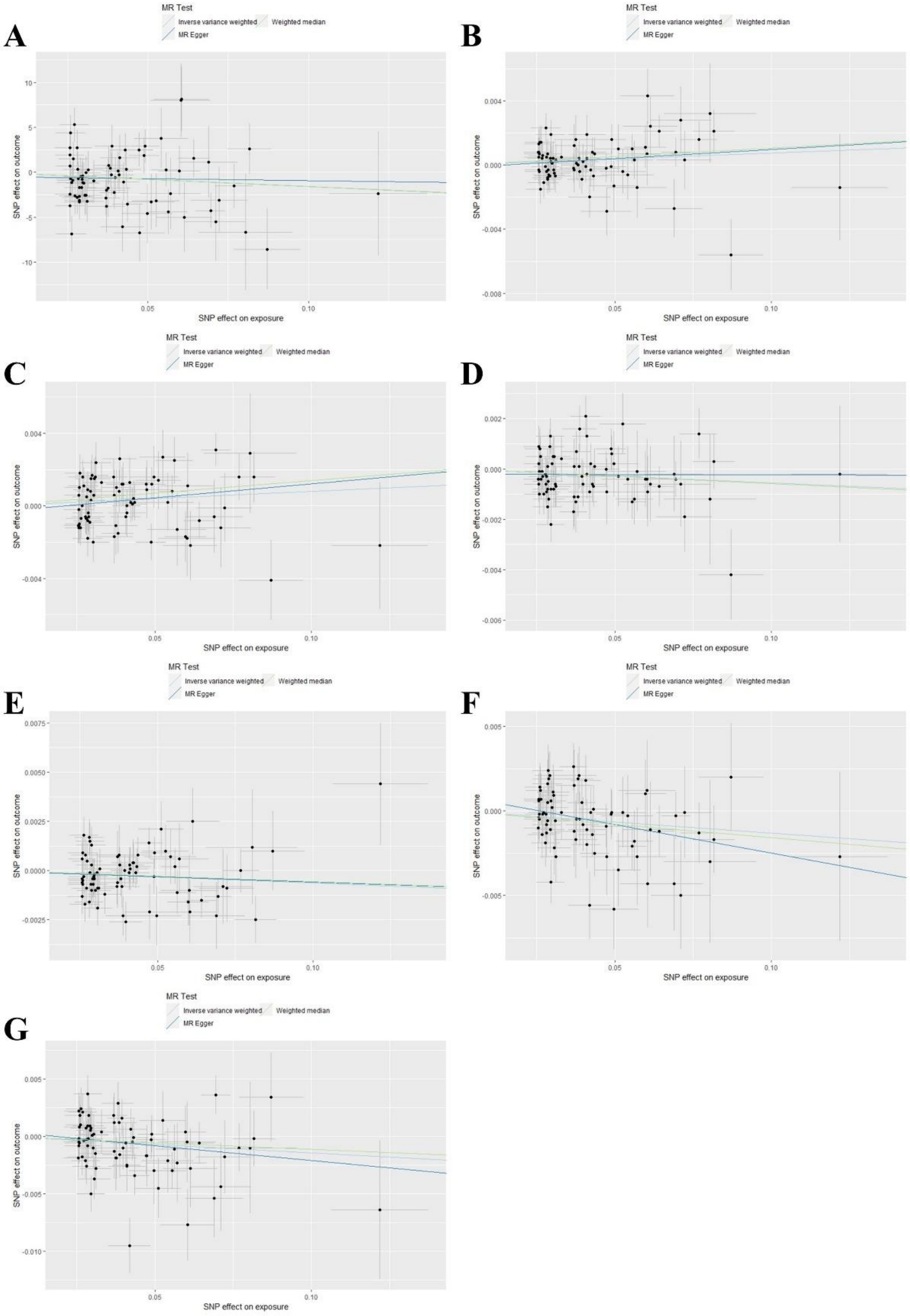

Figure S5. Funnel plots of nominally significant estimates from genetically predicted LIOP on A global weighted SA of the middle temporal; B. global weighted TH of the inferior temporal; C. global weighted TH of the insula; D. global weighted TH of the pars opercularis; E. global weighted TH of the superior temporal; F. global weighted TH of the transverse temporal; G. without global weighted TH of the transverse temporal.

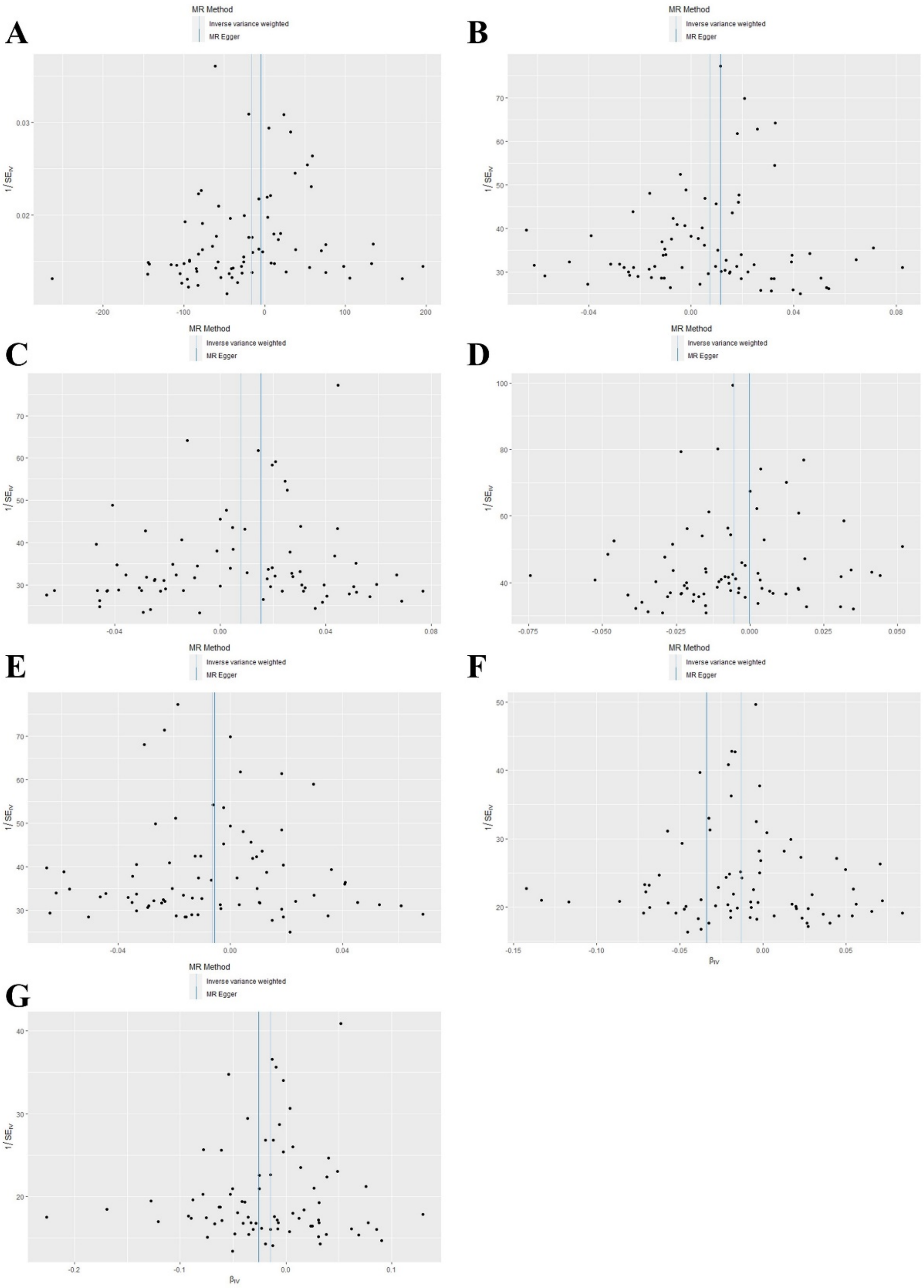

Figure S6. Leave-out plots of nominally significant estimates from genetically predicted LIOP on A global weighted SA of the middle temporal; B. global weighted TH of the inferior temporal; C. global weighted TH of the insula; D. global weighted TH of the pars opercularis; E. global weighted TH of the superior temporal; F. global weighted TH of the transverse temporal; G. without global weighted TH of the transverse temporal.

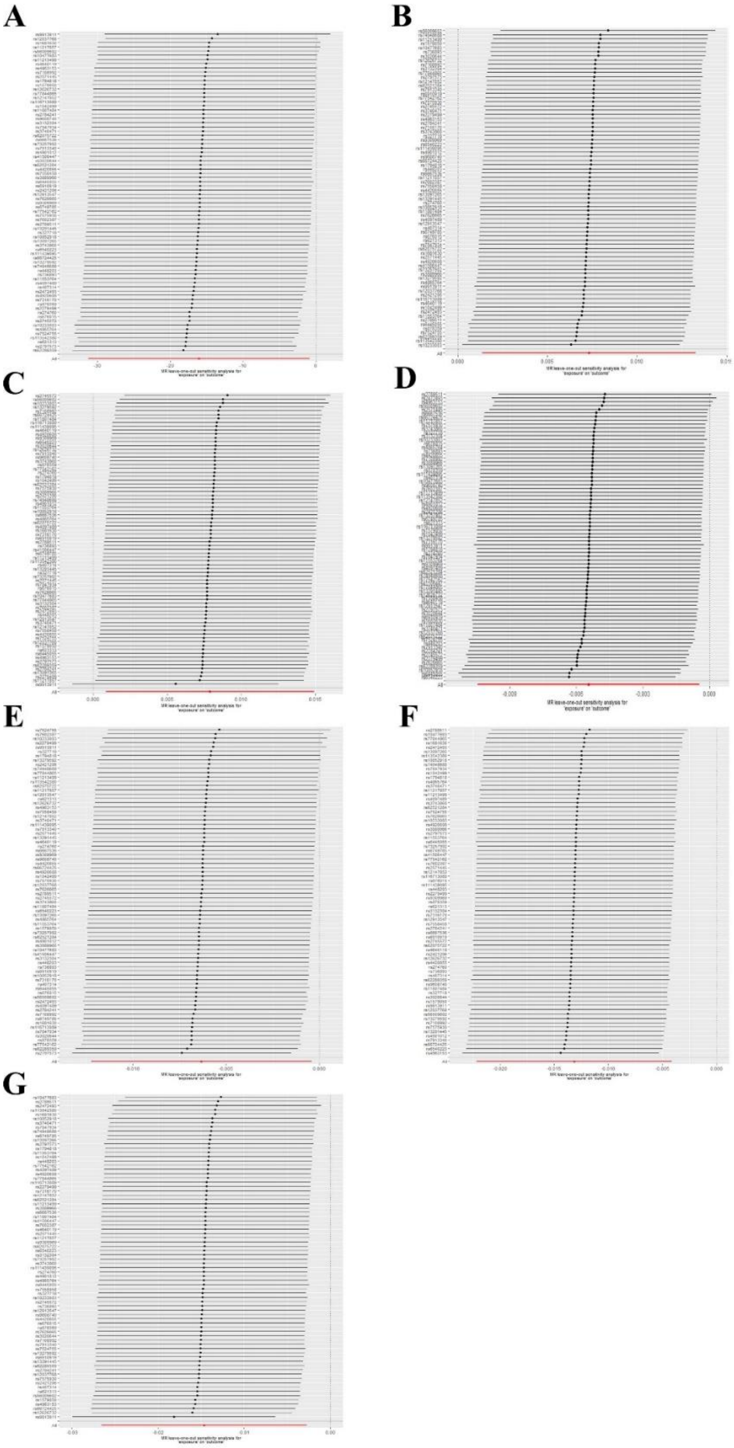

Figure S7. Scatter plots of nominally significant estimates from genetically predicted RIOP on A global weighted SA of the bankssts; B. global weighted SA of the middle temporal;C. global weighted SA of the parsorbitalis; D. global weighted TH of the inferior temporal; E. global weighted TH of the insula; F. global weighted TH of the superior temporal; G. global weighted TH of the transverse temporal. H. without global weighted SA of the bankssts; I. without global weighted SA of the middle temporal; J. without global weighted SA of the temporal pole; K. without global weighted TH of the transverse temporal.

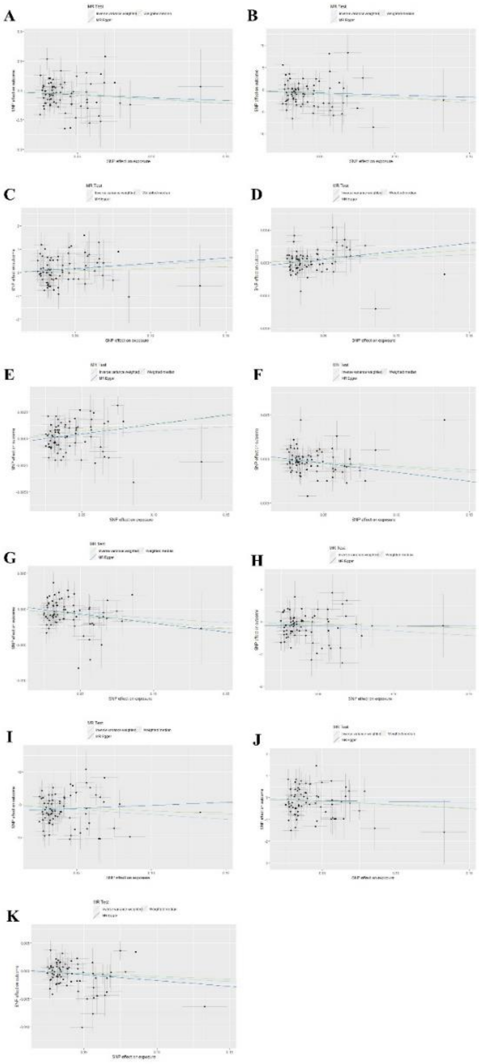

Figure S8. Funnel plots of nominally significant estimates from genetically predicted RIOP on A global weighted SA of the bankssts; B. global weighted SA of the middle temporal;C. global weighted SA of the parsorbitalis; D. global weighted TH of the inferior temporal; E. global weighted TH of the insula; F. global weighted TH of the superior temporal; G. global weighted TH of the transverse temporal. H. without global weighted SA of the bankssts; I. without global weighted SA of the middle temporal; J. without global weighted SA of the temporal pole; K. without global weighted TH of the transverse temporal.

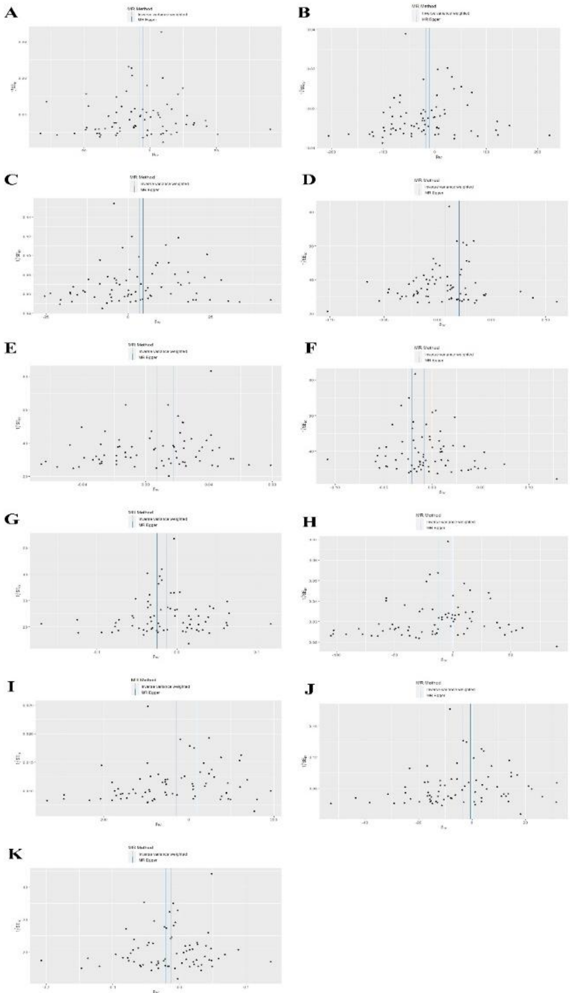

Figure S9. Leave-out plots of nominally significant estimates from genetically predicted RIOP on A global weighted SA of the bankssts; B. global weighted SA of the middle temporal; C. global weighted SA of the parsorbitalis; D. global weighted TH of the inferior temporal; E. global weighted TH of the insula; F. global weighted TH of the superior temporal; G. global weighted TH of the transverse temporal. H. without global weighted SA of the bankssts; I. without global weighted SA of the middle temporal; J. without global weighted SA of the temporal pole; K. without global weighted TH of the transverse temporal.

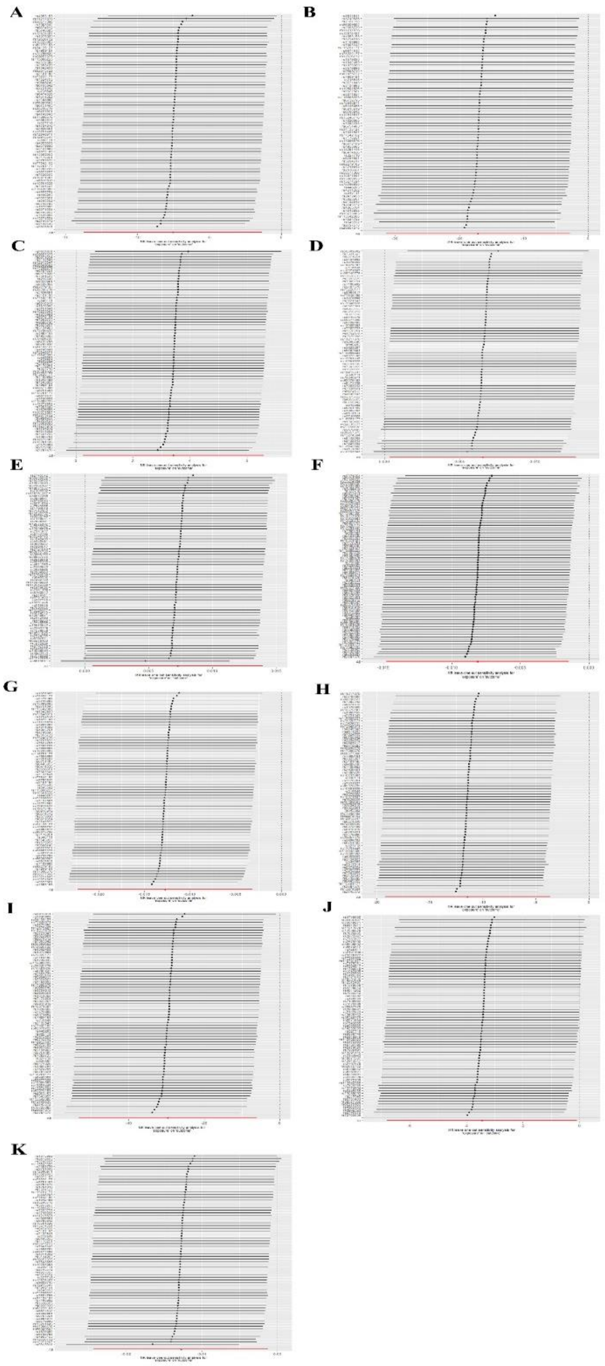

Figure S10. Scatter plots of nominally significant estimates from genetically predicted CDR on A global weighted SA of the medial orbitofrontal; B. global weighted SA of the middle temporal;C. global weighted TH of the lateral orbitofrontal; D. global weighted TH of the paracentral; E. global weighted TH of the postcentral; F. without global weighted TH of the entorhinal; G. without global weighted TH of the lateral orbitofrontal; H. without global weighted TH of the paracentral.

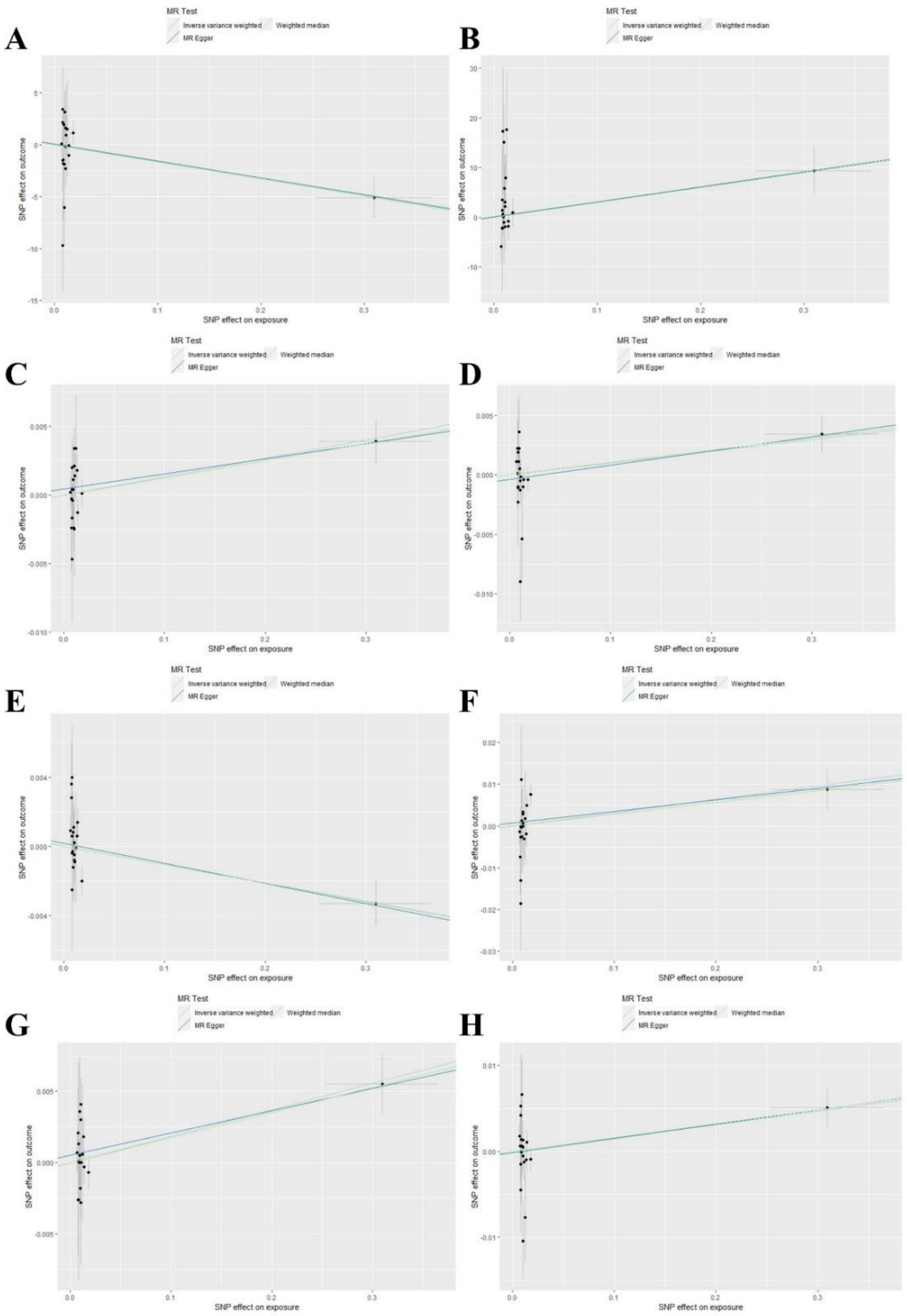

Figure S11. Funnel plots of nominally significant estimates from genetically predicted CDR on A global weighted SA of the medial orbitofrontal; B. global weighted SA of the middle temporal;C. global weighted TH of the lateral orbitofrontal; D. global weighted TH of the paracentral; E. global weighted TH of the postcentral; F. without global weighted TH of the entorhinal; G. without global weighted TH of the lateral orbitofrontal; H.without global weighted TH of the paracentral.

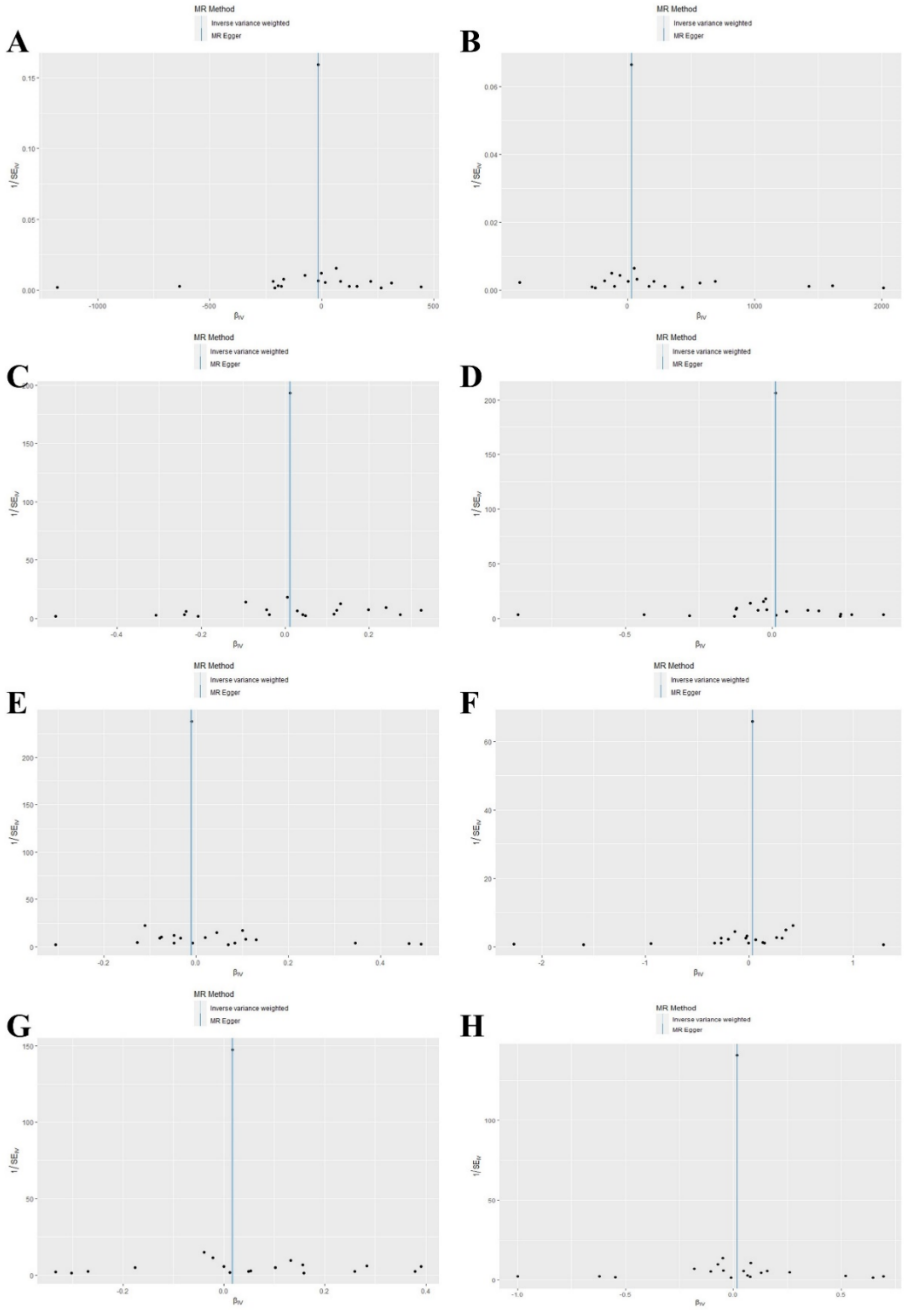

Figure S12. Leave-out plots of nominally significant estimates from genetically predicted CDR on A global weighted SA of the medial orbitofrontal; B. global weighted SA of the middle temporal; C. global weighted TH of the lateral orbitofrontal; D. global weighted TH of the paracentral; E. global weighted TH of the postcentral; F. without global weighted TH of the entorhinal; G. without global weighted TH of the lateral orbitofrontal; H. without global weighted TH of the paracentral.

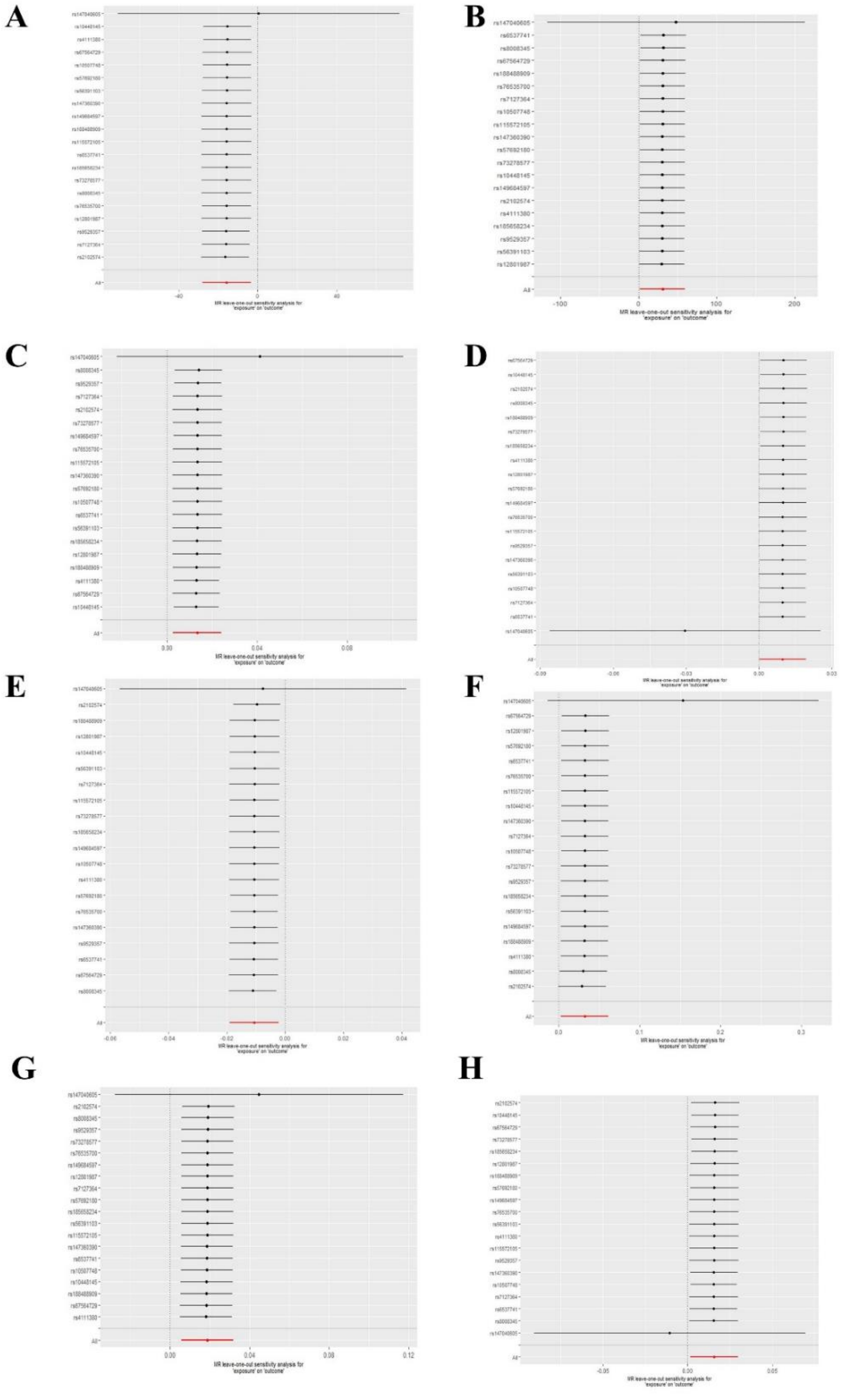

Figure S13. Scatter plots of nominally significant estimates from genetically predicted visual defects on A global weighted SA of the insula; B. global weighted TH of the superior temporal; C. global weighted TH of the temporal pole; D. without global weighted TH of the temporal pole.

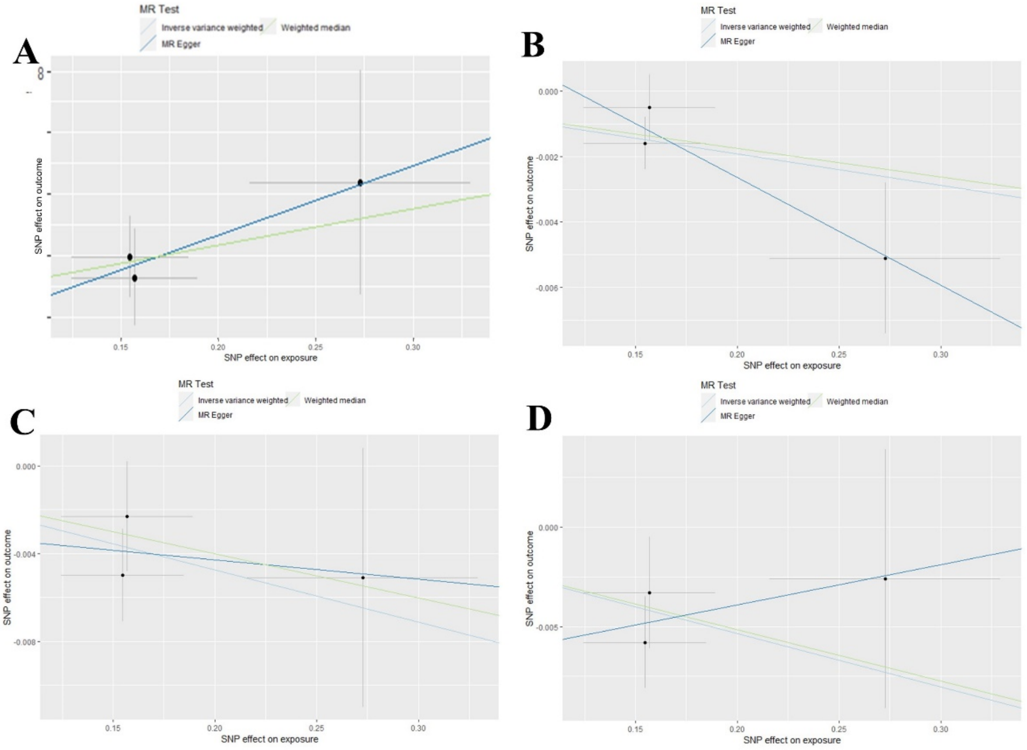

Figure S14. Funnel plots of nominally significant estimates from genetically predicted visual defects on A global weighted SA of the insula; B. global weighted TH of the superior temporal; C. global weighted TH of the temporal pole; D. without global weighted TH of the temporal pole.

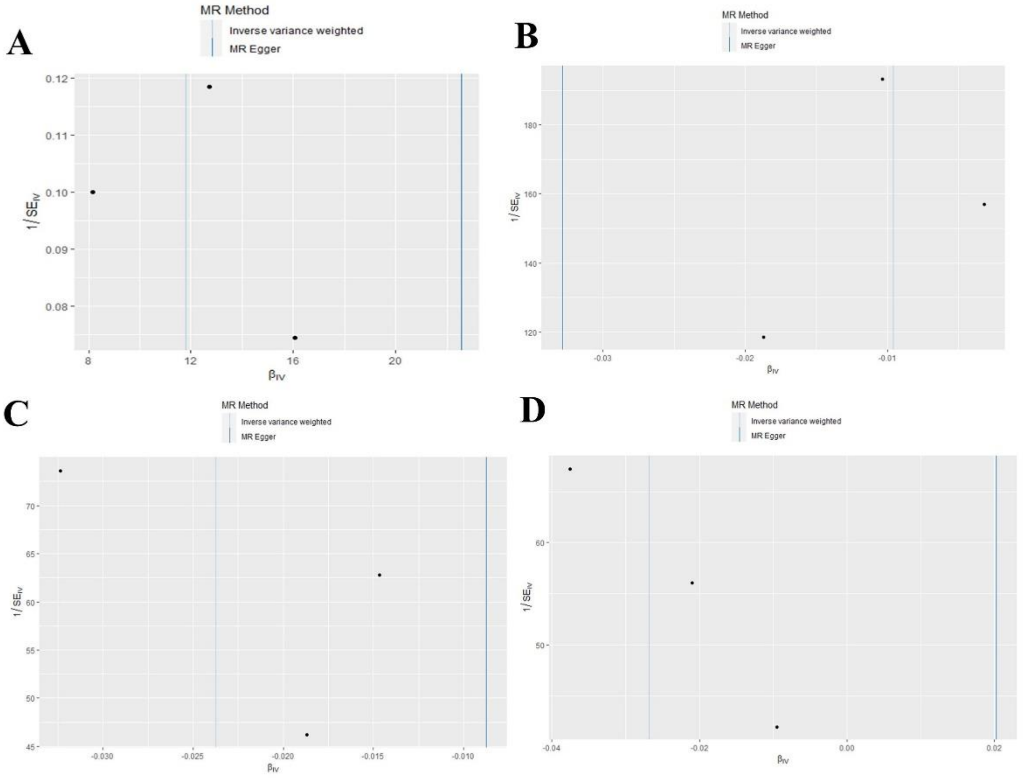

Figure S15. Leave-out plots of nominally significant estimates from genetically predicted visual defects on A global weighted SA of the insula; B. global weighted TH of the superior temporal; C. global weighted TH of the temporal pole; D. without global weighted TH of the temporal pole.

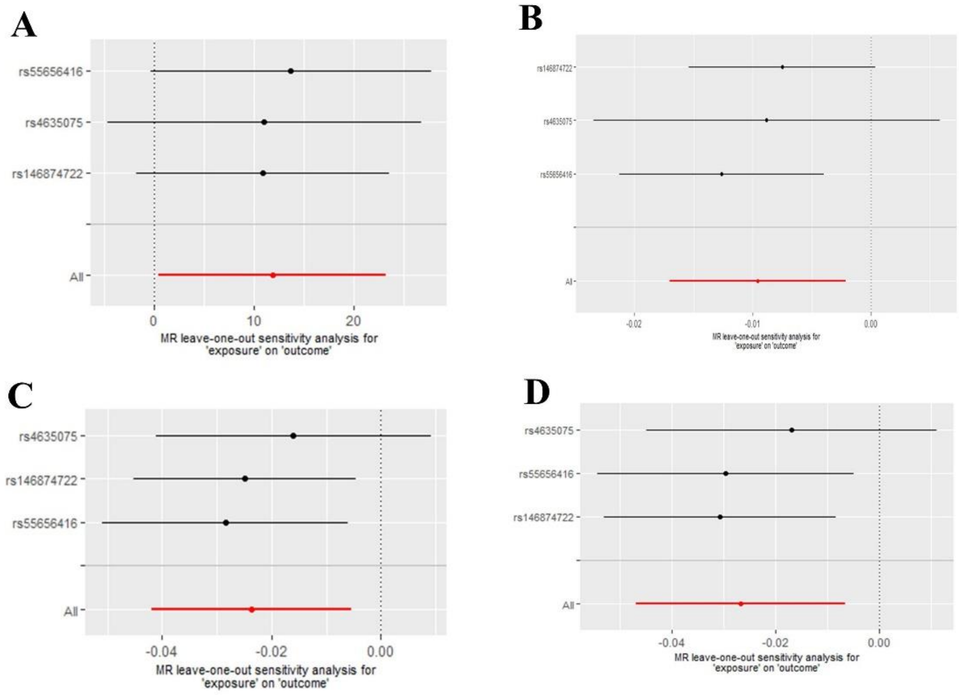

Figure S16. Scatter plots of nominally significant estimates from genetically predicted RNFL on A global weighted SA of the pericalcarine; B. without global weighted TH of the caudal anterior cingulate; C. without global weighted SA of the cuneus; D. without global weighted SA of lingual; E. without global weighted SA of the pericalcarine; F. global weighted SA of the cuneus; G. global weighted SA of the lingual.

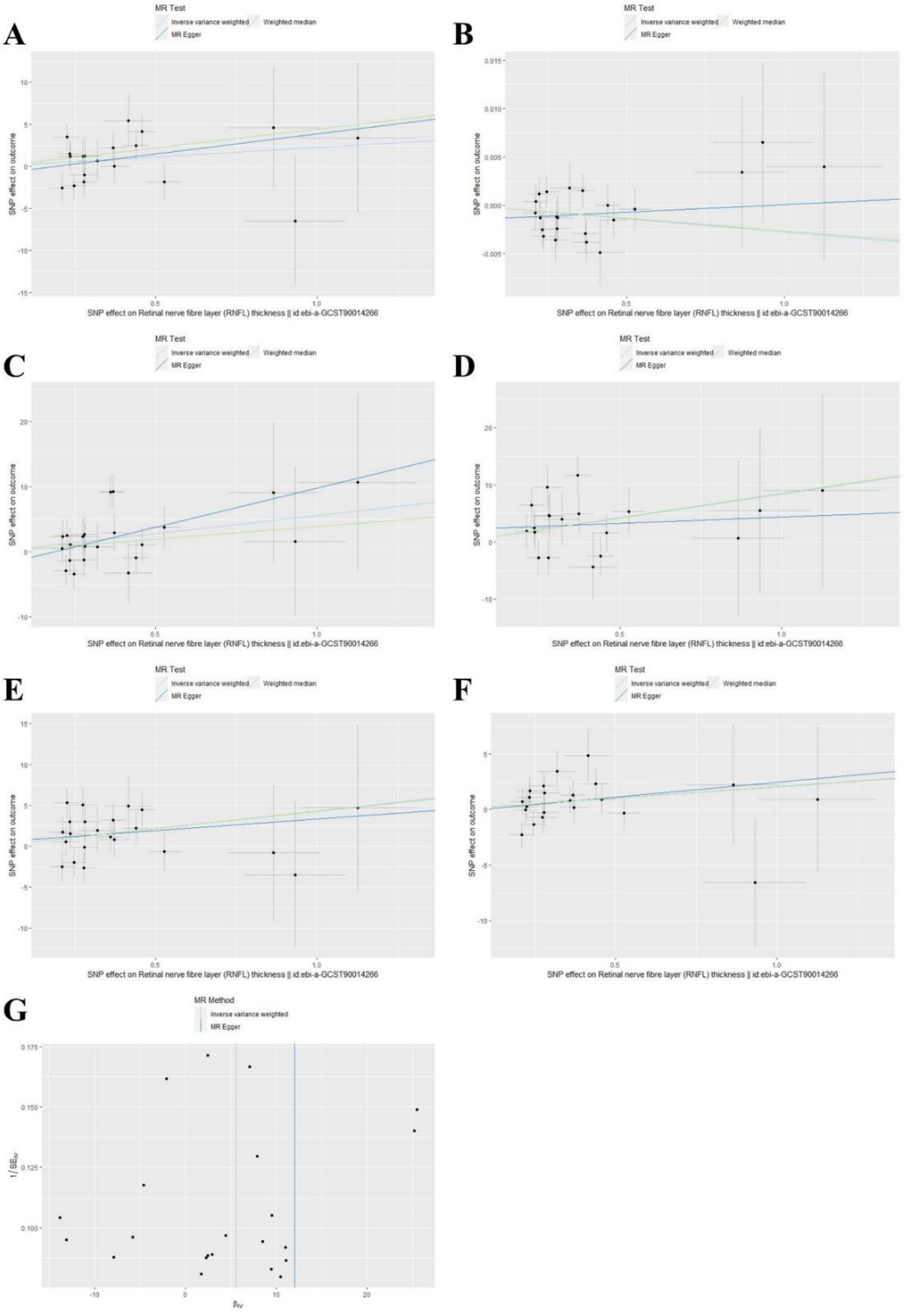

Figure S17. Funnel plots of nominally significant estimates from genetically predicted RNFL on A global weighted SA of the pericalcarine; B. without global weighted TH of the caudal anterior cingulate; C. without global weighted SA of the cuneus; D. without global weighted SA of lingual; E. without global weighted SA of the pericalcarine; F. global weighted SA of the cuneus; G. global weighted SA of the lingual.

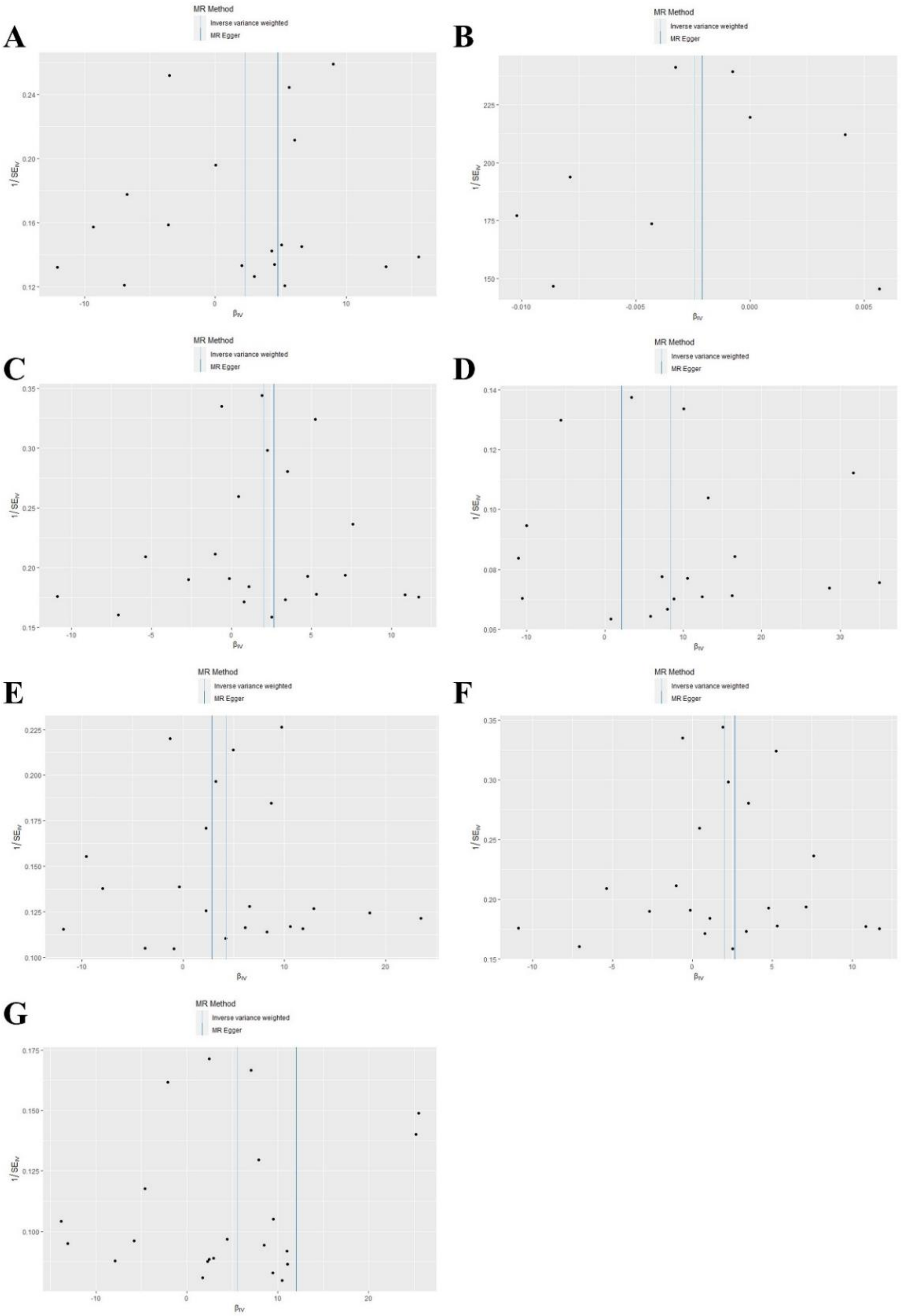

Figure S18. Leave-one-out plots of nominally significant estimates from genetically predicted RNFL on A global weighted SA of the pericalcarine; B. without global weighted TH of the caudal anterior cingulate; C. without global weighted SA of the cuneus; D. without global weighted SA of lingual; E. without global weighted SA of the pericalcarine; F. global weighted SA of the cuneus; G. global weighted SA of the lingual.

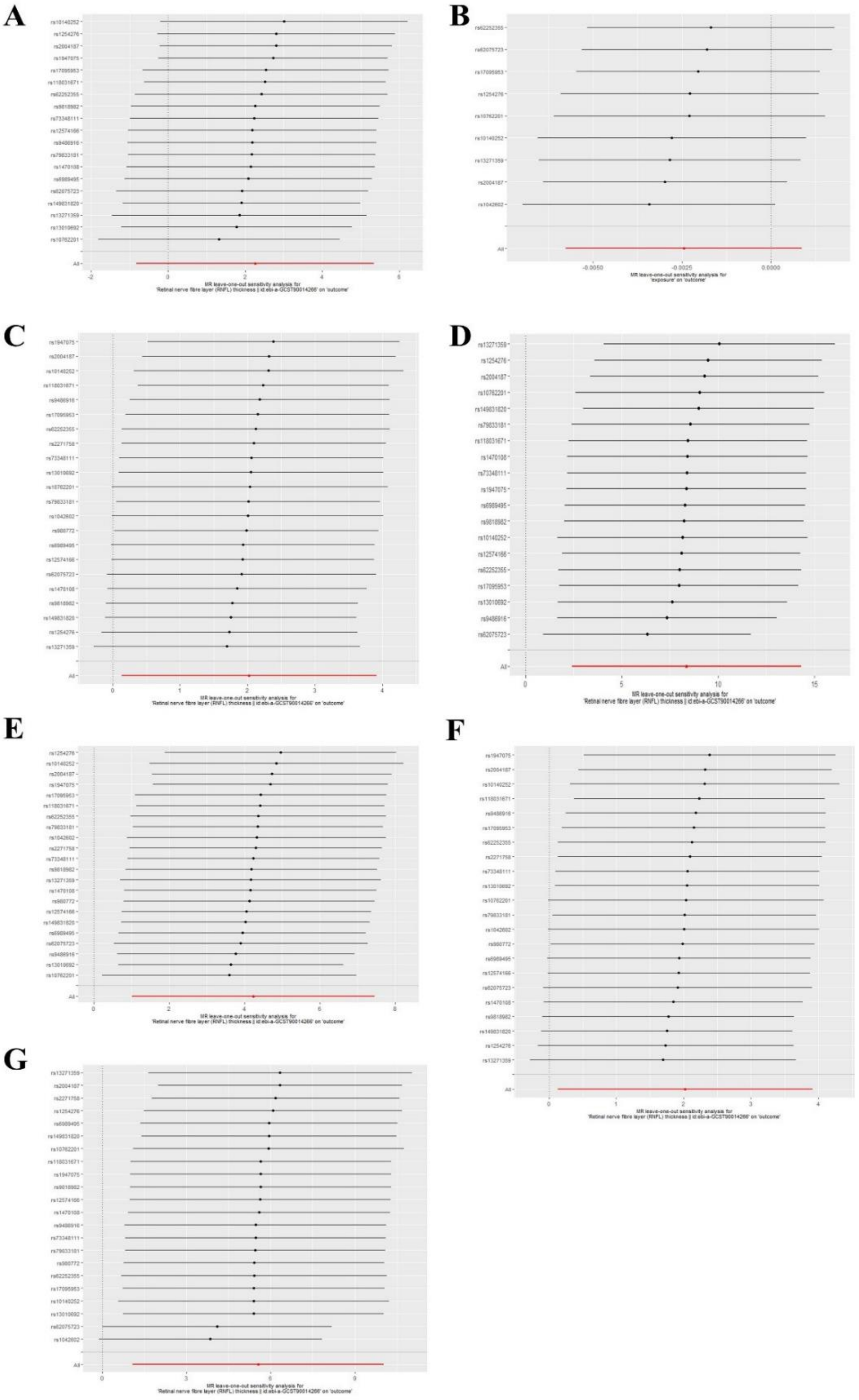

Supplement: Supplementary file 2 [file medi-104-e44416-s002.pdf]
